# Reversible Cationic Glycopolymer Hydrogels for RNA Delivery in Ticks

**DOI:** 10.1021/acspolymersau.6c00064

**Published:** 2026-06-14

**Authors:** Zoe A. B. Lequeux, Kundu Thapa, Musa Rabiu, Alec N. Simmons, Laila C. W. Peery, John F. Searles, Shahid Karim, Sarah E. Morgan

**Affiliations:** † School of Polymer Science and Engineering, 5104 University of Southern Mississippi, 118 College Drive # 5050, Hattiesburg, Mississippi 39406, United States; ‡ School of Biological, Environmental, and Earth Sciences, University of Southern Mississippi, 118 College Drive #5018, Hattiesburg, Mississippi 39406, United States

**Keywords:** self-healing, gene-knockdown, glycopolymer, hydrogels, cationic

## Abstract

RNA interference (RNAi) offers a promising approach for vector control in species such as the lone star tick (Amblyomma americanum), but current delivery methods, including microinjection and soaking, are invasive and inefficient for large-scale application. In this work, we developed a series of novel physically cross-linked cationic glycopolymer hydrogels for noninvasive double-stranded RNA (dsRNA) delivery and evaluated their rheological properties, release behavior, and in vivo performance. Rheological characterization demonstrated shear-thinning and self-healing behavior conducive to sprayable applications, while release studies revealed tunable cargo delivery dependent on glycomonomer stereochemistry and cationic content. In vivo studies showed that cationic terpolymer hydrogels successfully traversed the peritrophic membrane and enabled tissue-specific gene silencing, including in ovarian tissue. These findings establish a promising platform for sprayable RNAi delivery and provide a framework for the design of targeted gene delivery systems.

## Graphical abstract

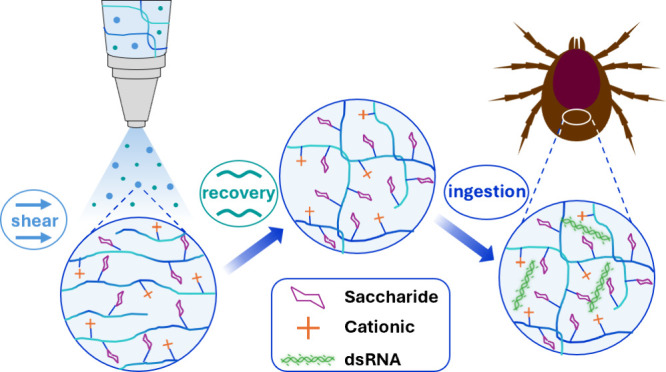

## Introduction

The lone star tick is a medically significant vector that transmits a variety of pathogenic bacteria, parasites, and viruses affecting both humans and livestock. Notably, this vector transmits Alpha-gal syndrome 9 (AGS), a red meat allergy triggered by IgE antibodies against galactose-α-1,3-galactose after bites, which has been estimated to exceed 450,000 cases in the U.S. between 2010 and 2022.^1–3^ In addition, the lone star tick is a major vector of *Ehrlichia chaffeensis*, the agent of human monocytic ehrlichiosis, and has also been associated with *Borrelia lonestari*, a bacteria that causes a rash and flu-like symptoms, and is sometimes confused with Lyme disease.^4,5^ Lyme disease is the most common tick borne disease, and is transmitted by the black-legged tick.^6^ Vector-borne pathogen transmission occurs during blood-feeding stages after prolonged host attachment, where the risk of certain infections, such as Lyme disease, can increase to 50% after 24–36 h of attachment.^7,8^ Unfortunately, there are currently no effective methods to prevent tick attachment or subsequent pathogen transmission. In addition, traditional population control methods, such as acaricides, are becoming less effective due to resistance development. RNAi, particularly dsRNA-mediated gene silencing, has shown promise in combating pathogen transmission by targeting essential genes. RNAi has also been shown to be a particularly useful technique for understanding pathogen-vector processes in ticks.^9–11^ Delivery of dsRNA in ticks is most commonly achieved by microinjection, which is effective but can lead to increased stress response and high mortality rates.^12–16^ Moreover, rapid in vivo breakdown of dsRNA, poor cellular uptake, and the lack of tissue-specific delivery further limit the potential of RNAi in ticks. Polymeric systems, with their high tunability and potential to incorporate multifunctionality and stimuli-responsive moieties, provide the potential to protect the naked dsRNA, target delivery, and release it in the desired time frame.^17,18^

The use of cationic polyelectrolytes as nonviral transfection agents has become a promising approach to enhance the cellular uptake of dsRNA in mammalian and insect cell studies.^19–21^ However, high cationic charge densities tend to be highly cytotoxic. The incorporation of saccharide functionalities into cationic polyelectrolytes has been shown to reduce the cytotoxic response due to the structural similarity to naturally occurring polysaccharides and glycoproteins.^22,23^ Previous work in our lab by Stockmal et al., demonstrated that cationic glycopolymer (synthetic polymer with pendent saccharide moieties) complexes significantly increased the RNAi response during tick in vitro studies compared to naked RNA strands, while also exhibiting lower cytotoxicity than commercially available transfection agents.^24^ In addition, it was found that glycomonomer type played a crucial role in cellular uptake: galactose-based complexes aided uptake, whereas glucose-based complexes did not. While these results are encouraging in vitro, translation to in vivo applications requires delivery methods that can replace microinjection.

Noninvasive RNAi delivery strategies, such as soaking or capillary feeding, have demonstrated potential and could be further improved by the use of hydrogels as carriers.^25,26^ Hydrogel-based delivery systems provide prolonged, tunable release of biomolecules while shielding dsRNA from deterioration. In particular, physically cross-linked hydrogels have become increasingly attractive for localized delivery as they are formed through noncovalent interactions such as hydrogen bonding, ionic interactions, π–π stacking, and hydrophobic associations, resulting in dynamic, reversible networks.^27–29^ These reversible bonds open the pathway to the development of sprayable hydrogels, which could significantly expand the applicability of hydrogel-based RNAi systems. For effective spraying, hydrogels must exhibit shear-thinning behavior and rapid viscoelastic recovery.^30,31^ These properties allow the hydrogel to withstand the high shear stresses generated during spraying while maintaining functional performance upon application.

Previous work in our lab by Wang et al. has introduced novel glycopolymer physically cross-linked hydrogels.^32^ These materials exhibited strong shear-thinning behavior and rapid recovery, making them well-suited for sprayable delivery systems. Incorporation of a galactose-based monomer improved both the recovery and adhesive properties of the hydrogels. Comonomer selection (*N*-isopropylacrylamide (NiPAm) vs *N*-hydroxyethyl acrylamide (HEAm) was also shown to influence the release of small molecule dyes, indicating the potential for tunable drug release through polymer structure. Despite advances in hydrogel-based delivery systems, a gap remains in the literature regarding sprayable cationic glycopolymer hydrogels and their in vivo evaluation for RNAi applications.

Building on the findings from Stockmal and Wang, we synthesized a series of novel, physically cross-linked cationic terpolymer glycohydrogels of different compositions via free radical polymerization and compared them to a cationic copolymer control. In addition to the glycomonomers, we selected NiPAm and *N,N*-dimethylaminopropylacrylamide (DMAPAm) as comonomers for their biomedical relevance and potential stimuli-responsive properties. NiPAm is extensively studied in hydrogels for its lower critical solution temperature (LCST) transition at 37 °C, which could enable a burst release of dsRNA during tick feeding.^33,34^ DMAPAm, a cationic monomer, was selected for its pH-responsive properties and ability to complex with anionic dsRNA strands, potentially enhancing RNAi.^24,35^ Defining the effects of glycomonomer type and structure on hydrogel rheology, dsRNA release, and in vivo performance will provide a greater understanding of parameters for the design of sprayable gene delivery vehicles.

## Experimental Section

### Materials

*N*-(2-Hydroxyethyl)­acrylamide (HEAm, 97%), potassium persulfate (KPS, >99.0%), *N*-isopropylacrylamide (NiPAm, >99.0%), *N*,*N*,*N*′,*N*′-tetramethylethylenediamine (TEMED, >99.5%), silver triflate (AgOTf, >98%), acetobromo-α-d-galactose (AcGalBr, >93%), acetobromo-α-d-glucose (AcGlcBr, >93%), methyl orange (MO, dye content 96%), acetonitrile (MeCN, >99.8%), deuterium oxide-*d*_2_ (D, 99.9%), ribonucleoside triphosphates (NTPs), and dimethyl sulfoxide-*d*_6_ (D, 99.9%) (DMSO-*d*_6_) were purchased from Sigma-Aldrich. Phosphate buffer saline (PBS) tablets, *N*,*N*-dimethylformamide (DMF), methanol (CH_3_OH), sodium methoxide methanol solution (30 wt %), hydrochloric acid (HCl, >36.5% v/v), potassium hydroxide (KOH, >85%), dichloromethane (DCM), molecular sieves (4 Å, powder), glacial acetic acid (AcOH, >99.5%), chloroform-*d* (D, 99.8%), ethanol (>96%), sodium chloride (5M), sodium acetate (anhydrous, >99%), labeling buffer, DNA template, T7 RNA polymerase, nuclease-free water, and Nanodrop One were purchased from Thermo Scientific Chemicals. PCR Primers, DNase I, iScript cDNA synthesis kit, agarose gel, and Cystatin dsRNA were purchased from Bio-Rad. Calcium sulfate (anhydrous) was purchased from W.A. Hammond Drierite Company, Ltd. TRI reagent (TRIzol) was purchased from the Molecular Research Center, Inc. RNALater was purchased from Invitrogen. A T7 Quick High Yield RNA Synthesis Kit was purchased from New England Biolabs. A QIAquick PCR Purification Kit was purchased from QIAGEN. Dimethylaminopropylacrylamide (DMAPAm, >98%) was purchased from TCI Chemicals. All chemicals were used without further purification. The deacetylated glycomonomers 2′-acrylamidoethyl-β-d-galactopyranoside (GalEAm) and 2′-acrylamidoethyl-β-d-glucopyranoside (GlcEAm), were synthesized and purified according to a reported procedure.^36^ Spectra/Por 6 dialysis membrane prewetted RC tubing with a molecular weight cut off of 2 kDa was used for purification. Transwell culture plates (Costar 3413, 24-well, 6.4 mm with 0.4 μm pore size filters) were purchased from Corning Incorporated.

### Glycohydrogel Synthesis

Copolymer hydrogel networks composed of glycomonomer, DMAPAm, and NiPAm were synthesized via free radical polymerization at room temperature, without inert gas purging before or during the reaction. Monomer solutions and the KPS stock solution were prepared in DI water and stirred for 30 min under ambient conditions until fully dissolved. The TEMED stock solution was prepared separately in DI water and manually shaken to ensure homogeneity. KPS was then added to the monomer solution and vortexed for 10 s. Immediately after, TEMED was added, followed by an additional 10 s of vortexing. The resulting solution was left undisturbed at room temperature for 24 h to allow polymerization to proceed. The total concentrations of monomer (1 M), KPS (2 mol %), and TEMED (1 mol %) were kept constant across all polymerizations. Copolymerization monomer feed ratios are listed in Table 1, and polymer structures are shown in Figure 1b.

**Figure 1:**
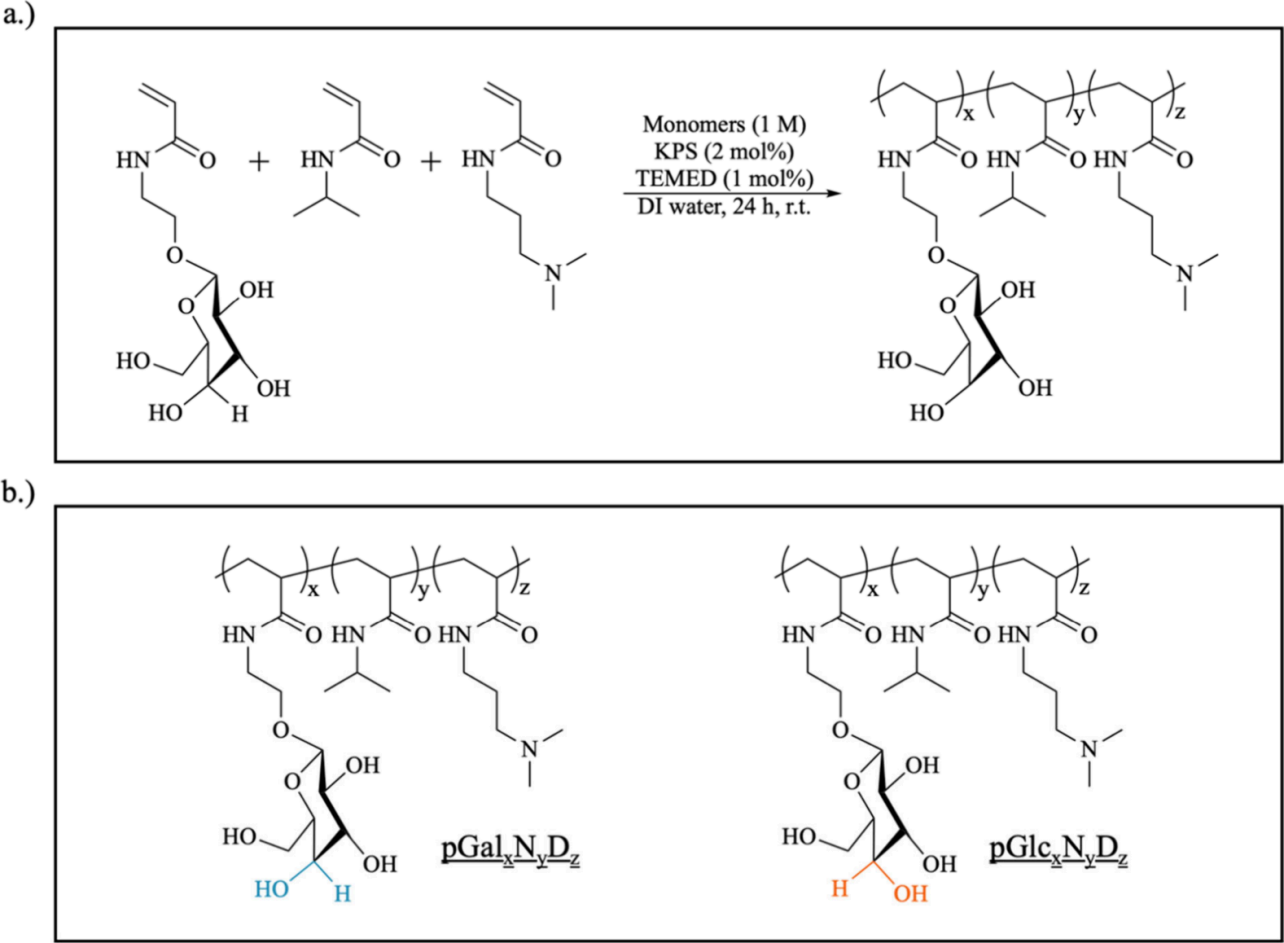
a) Reaction scheme of the GalEAm containing terpolymer. b) Chemical structures of GalEAm and GlcEAm-based terpolymer hydrogels. A green polymerization method was employed, using water, room temperature, and ambient environment.

**Table 1:** Hydrogel Compositions (mol %)

Hydrogel	Glyco (feed)	Glyco (exp)^a^	NiPAm (feed)	NiPAm (exp)^a^	DMAPAm (feed)	DMAPAm (exp)^a^
pN_53_D_47_			50	53.3 ± 1.2	50	46.7 ± 1.2
pGlc_39_N_39_D_22_	35	39.0 ± 1.6	50	39.3 ± 1.2	15	21.7 ± 0.5
pGlc_22_N_41_D_37_	15	22.0 ± 1.6	50	41.3 ± 0.5	35	37.0 ± 1.4
pGal_31_N_45_D_24_	35	31.3 ± 2.6	50	44.7 ± 1.9	15	24.0 ± 1.4
pGal_16_N_45_D_39_	15	16.0 ± 0.0	50	44.7 ± 1.2	35	39.3 ± 0.5

a Hydrogel composition determined by comparing integrations of specific comonomer protons using 400 MHz ^1^H NMR spectroscopy in DMSO-*d*_6_, relaxation delay = 5 s. Values represent mean ± 1 standard deviation (*n* = 3).

### Sample Preparation

Multiple hydrogel sample types were prepared for specific tests. As-polymerized (AP) samples refer to the unpurified hydrogel reaction mixture. The initial solids content of the AP samples is estimated to be 18.6 wt % based on the composition of the reaction mixture. AP samples were used for rheological characterization, and a portion of each AP hydrogel was purified by dialysis against DI water for 3 days, then freeze-dried for structural characterization. Swollen Purified (SPure) samples refer to AP hydrogels that were first purified via dialysis against DI water for 3 days, followed by swelling in excess DI water for 5 days at room temperature to reach the equilibrium swelling point. Excess water was then decanted, and the samples were gently blotted dry.

The hydrogel stimulus response was determined by adjusting the pH of the SPure samples. HCl (0.01 M) and KOH (0.01 M) solutions were used to adjust the SPure samples to a pH of 3 (SPure_pH3) and a pH of 10 (SPure_pH10), respectively. After the pH was adjusted, the samples were left to equilibrate for 5 days at room temperature. SPure_pH3 samples were used for rheological characterization and diffusion studies.

Cystatin dsRNA-loaded hydrogels (SPure_RNA) were prepared for rheological studies. The amine to phosphate group (N:P) ratio was maintained at 25 for all hydrogel compositions tested. RNA was loaded into the hydrogel by injecting the hydrogel with a specific volume of RNA solution (0.4 mg/mL) in DI water to achieve the desired N:P ratio. Samples were left overnight to equilibrate before testing.

### Thermogravimetric Analysis (TGA)

The solid content of the swollen hydrogel samples was determined by the residual mass ratio obtained using a TA Instruments Discovery TGA 550 (New Castle, DE) under nitrogen using platinum HT pans. The changes in weight of the swollen hydrogel were monitored by heating from room temperature to 180 °C with a temperature ramp of 10 °C min^–1^. Each sample was measured in triplicate.

### NMR Spectroscopy

^1^H NMR spectroscopy was performed to determine both the mol % of unreacted monomer and the molar ratio of monomers in the polymer chain. The spectra were acquired using a 400 MHz Bruker Avance III spectrometer (TopSpin 3.1p17), with DMSO-*d*_*6*_ utilizing 128 scans and a relaxation delay = 5 s. To quantify the mol % of unreacted monomer, NMR analysis was conducted on AP hydrogels without purification or drying. The integral of the internal standard, DMF (7.95 ppm), was compared to the two protons corresponding to the acrylamide handle (6.22–6.03 ppm) of the monomers. For terpolymer composition determination (GalEAm or GlcEAm-coNiPAm-coDMAPAm), NMR spectroscopy was performed on purified, freeze-dried polymers. The composition was calculated by comparing the integrals of the saccharide acetyl proton (4.15 ppm) of the glycomonomers with the six protons of the NiPAm methyl groups (1.06 ppm) and the two protons from the DMAPAm monomer (1.55 ppm). The NiPAm-*co*-DMAPAm copolymer composition was determined by comparing the integrals of the two protons from the DMAPAm monomer (1.55 ppm) with the six protons of the NiPAm methyl groups (1.06 ppm). All NMR spectra were processed, deconvoluted, and analyzed using Mnova software. Each sample was measured in triplicate.

### Aqueous Size Exclusion Chromatography with Multiangle Laser Light Scattering (ASEC-MALLS)

Weight-average (*M*_w_) and number-average (*M*_n_) molecular weights of the freeze-dried, purified GalEAm homopolymer (pGal) were determined using ASEC-MALLS on an Agilent 1260 Infinity II LC system equipped with a PL aquagel MIXED-OH column, a DAWN HELIOS-II light scattering detector (λ = 633 nm, Wyatt Technology Inc.), and an Optilab T-rEX refractometer (Wyatt Technology Inc.). TRIS buffer (pH = 8.0) with 0.01% (w/v) NaN_3_ was used as the eluent at a flow rate of 0.5 mL/min with a sample concentration of 1 mg/mL and an injection volume of 100 μL. The refractive index increment (d*n*/d*c*) value of the pGal was determined by a Reichert ARAIS 500 refractometer to be 0.145 mL/g. ASEC-MALLS spectra were analyzed using Wyatt ASTRA SEC/LS software (version 7.1.4.8) to determine the *M*_n_, and *M*_w_.

### LCST Determination

The LCST of the SPure hydrogels was probed by heating the hydrogels to 37 or 50 °C and maintaining the set temperature for 5 min. The hydrogel samples were then removed and inverted for 30 s before a picture was taken. Pictures were taken on an iPhone 14 Pro in portrait mode.

### Rheology Characterization

The viscoelastic properties and self-healing characteristics of the various hydrogel samples were evaluated using a strain-controlled ARES rheometer equipped with 25 mm parallel steel plates with a gap of 0.5 ± 0.05 mm, at 21 °C. Dynamic strain sweep experiments were conducted at an oscillatory frequency of 6.28 rad/s to determine the linear viscoelastic region (LVR). Small amplitude oscillatory shear (SAOS) tests were performed over a frequency sweep range of 1–100 rad/s at 10% strain (within the LVR) to obtain the storage modulus (*G*′), loss modulus (G″), and complex viscosity (η*). A power law model was used to describe the relationship between storage modulus (*G*′) and frequency (ω), shown in eq 2.1:

2.1 $$G^{\prime }=A^{\prime }\omega^{n\prime }$$

where *n*′ is the slope and log­(*A*′) is the intercept in the log–log plot, also described as the zero shear modulus (*G*′_0_).

The shear-thinning index (*n*) was calculated by fitting the η* data to the power law flow model using eq 2.2:

2.2 $$\eta =K{\mathrm{\gamma ̇}}^{n-1}$$

where η is apparent viscosity, *γ̇* is shear rate, and *K* is the consistency index.

The hydrogel mesh size (ξ) was calculated using elastic blob theory under the assumption of the affine network theory and a cubic lattice network.^37,38^ In this framework, the zero shear modulus is related to the number density of elastically active network strands according to eq 2.3:

2.3 $${G^{\prime }}_{0}=\rho_{el}k_{B}T$$

The mesh size was then approximated as the mean distance between elastic blobs using eq 2.4:

2.4 $$\xi ={\rho_{el}}^{-1/3}$$

where *ρ*_*el*_ is the number density of elastic blobs, *k*_B_ is the Boltzmann constant, *T* is temperature, and *G*′_0_ is the zero shear modulus calculated in eq 2.2.

The self-healing characteristics were evaluated through dynamic oscillatory rheological experiments. The samples underwent cyclic step strain experiments, alternating every 100 s between a low strain (10%) within the LVR and a high strain (700%) beyond the LVR to intentionally disrupt the gel network. This procedure was repeated for three cycles to assess the recovery of the hydrogel modulus. Each sample was measured in triplicate.

### Release Study

MO was used to determine the cumulative release profile of the SPure and SPure_pH3 hydrogels following a previously described procedure.^39^ Each sample was preweighed (∼100 mg), placed in a Transwell insert, and incubated with 20 μL of a 1 mg/mL MO solution for 24 h. One mL of DI water (release media) was added to the lower chamber, and periodically, 200 μL of the free liquid was removed for UV–vis analysis and replaced with an equivalent amount of fresh DI water. The MO concentration (C­(t)) was determined using a BioTek Synergy 2 microplate reader using a previously prepared calibration curve. The cumulative release of MO was calculated using eq 2.5:

2.5 $$\mathrm{Cumulative}\;\mathrm{Release}\;\left(\%\right)=\frac{C_{n}\times \nu_{0}+\Sigma_{1}^{n-1}C_{i}\left(t\right)\times \nu_{e}}{M_{\left(Loaded\right)}}\times 100$$

where *M*_(*Loaded*)_ is the initial mass of MO loaded, *v*_0_ is the total volume of release media, *v*_*e*_ is the volume of release media removed at each time period, *C*_*i*_(*t*) is the concentration of dye in the release media at time *t*, and *C*_*n*_ is the final concentration of dye in the release media.

### Ticks Acquisition and Maintenance

*Amblyomma americanum* (L.) ticks were obtained from Oklahoma State University’s tick rearing facility, where they are raised following the methods of Patrick and Hair.^40^ Unfed ticks were kept at 27–28 °C with 90% relative humidity under a 14 h light/10 h dark photoperiod before infestation on the sheep. Ticks were blood-fed on sheep and allowed either to feed to repletion or were partially fed and removed after 7 days, depending on the study. All animal studies were performed in accordance with protocols approved by the Institutional Animal Care and Use Committee (IACUC) at the University of Southern Mississippi (protocol # 15101501 and #15011402).

### Preliminary In Vivo Study

Three hydrogel compositions (pGal_41_N_59_, pGal_56_D_44_, and pGal_25_N_34_D_41_) were synthesized as described above, purified by dialysis against DI water, and subsequently freeze-dried. The hydrogels were reswollen in DI water containing food dye before use. Unfed female ticks were placed in sterile vials and left for 24–48 h in a desiccator containing anhydrous calcium sulfate. The desiccated ticks were then placed in Petri dishes with a small amount of the hydrogel-dye mixtures, allowed to imbibe, and monitored. Ticks were dissected to determine whether the hydrogel and associated dye passed through the peritrophic membrane.

### Tick Tissues Collection

Partially and fully blood-fed female adult ticks were dissected within 4 h of removal from the sheep. Midgut, salivary glands, and ovarian tissues were collected in ice-cold M-199 buffer.^41^ Following dissections, tissues were washed gently in the same ice-cold buffer and then preserved in RNAlater or TRIzol for later RNA extraction.

### Cystatin dsRNA Preparation

Total RNA was extracted from the dissected tissues of fully fed ticks following the TRIzol-chloroform separation and isopropanol precipitation methods with slight modification.^42^ Briefly, RNA pellets were washed in 75% ice-cold ethanol, air-dried, and resuspended in 30 μL nuclease-free water. The final RNA concentration and quality were checked on a nanodrop and were then stored at −80 °C until needed.^12,43^ Complementary DNA (cDNA) synthesis was performed from 1000 ng of isolated RNA as previously described.^44^ To synthesize cDNA, 2 μg of RNA was added to a 20 μL reaction of the iScript cDNA synthesis kit. The reverse transcription reaction was then heated in a Bio-Rad thermocycler under the following conditions: 5 min at 25 °C, 30 min at 42 °C, 5 min at 85 °C, followed by a hold at 4 °C. The resultant cDNA was diluted to a working concentration of 25 ng/μL with nuclease-free water and stored at −20 °C until use. Cystatin dsRNA was then produced by amplifying the cDNA using PCR containing T7 promoters for *A. americanum* midgut cystatin (Table S1). PCR was performed under the following conditions: 94 °C for 3 min, 94 °C for 30 s, 35 cycles of 60 °C for 30 s, 68 °C for 1 min, and 68 °C for 8 min, and a hold at 4 °C. The resulting Cystatin dsRNA was used in rheological studies.

### Galectin dsRNA Preparation

Synthesis of dsRNA and tick manipulations were performed following previously described methods.^1,44^ PCR products of galectin were purified using a QIAquick PCR Purification Kit and used as templates in secondary PCR with T7 promoter primers for dsRNA synthesis (Table S1). In vitro transcription was performed for galectin using the T7 Quick High Yield RNA Synthesis Kit. PCR amplification conditions were as follows: 94 °C for 3 min, 94 °C for 30 s, 35 cycles of 60 °C for 30 s, 68 °C for 1 min, and 68 °C for 8 min, and a hold at 4 °C. The resulting Galectin dsRNA was purified by ethanol precipitation, checked on a 2% agarose gel, and diluted to a concentration of 1 μg/μL in DI water.

### Galectin Silencing (RNA Interference) In Vivo Study

The effect of the hydrogels on Galectin dsRNA uptake and subsequent Galectin gene silencing was evaluated using four groups of 20 unfed female ticks, each exposed to a specific treatment. The treatments included a control of DI water without dsRNA and three RNA-loaded hydrogels at a concentration of 1 μg/μL. The selected hydrogel formulations (pN_53_D_47_, pGlc_39_N_39_D_22_, and pGal_31_N_45_D_24_) were chosen based on prior rheological characterization and release profile results. Ticks were separated into groups, placed in sterile vials, and desiccated for 24–48 h in a chamber containing anhydrous calcium. Following desiccation, ticks were placed in Petri dishes with their respective treatment solutions, allowed to imbibe the solution, and monitored. Ticks were then placed on sheep and allowed to partially feed in the presence of males. Tissue samples were collected from partially fed ticks and analyzed to assess Galectin gene silencing. Transcriptional expression was estimated by ΔΔCt method in Bio-Rad Software (Bio-Rad CFX manager V 3.1), and 2-fold differences with p-values of <0.05 were considered significant between the two groups. The data were presented as mean ± the standard error of the mean (SEM).

## Results and Discussion

### Structural Characterization

A series of physical hydrogels containing thermally responsive NiPam, pH-responsive DMAPAm, and acrylamide monomers with either β-d-galactose (Gal) or β-d-glucose (Glc) pendant groups (Figure 1) were prepared by aqueous ambient free radical polymerization in triplicate as described in the experimental section. Hydrogel composition was determined by ^1^H NMR (Figure S1), and the naming convention reflects the mean molar incorporation of comonomers (e.g., pGal_31_N_45_D_24_ indicates 31 mol % Gal, 45 mol % NiPAm, and 24 mol % DMAPAm). A 50% feed ratio of NiPAm was employed, with DMAPAm feed of 15, 35, or 50%. Hydrogel feed ratios and compositions are summarized in Table 1.

NiPAm is incorporated at a slightly higher than feed ratio in the copolymer, but at a lower than feed ratio in the terpolymers, particularly for Glc-containing systems. DMAPAm is incorporated at a slightly higher than feed ratio in the terpolymers, but no significant difference is observed for relative incorporation in Glc or Gal systems. Glc is incorporated at statistically significant higher ratios than Gal. The difference in glycomonomer incorporation is likely due to a difference in monomer reactivity stemming from the saccharide ring stereochemistry. It has been shown in other radical-mediated polymerizations that Glc has a shorter induction time and a higher reaction rate than Gal.^24,45^ Similarly, the reactivity of NiPAm was shown to be affected by saccharide structure in copolymerization with glycomonomers, which may explain the differences in NiPAm incorporation seen in the Gal and Glc terpolymers.^46^ The amount of unreacted monomer was approximated by performing ^1^H NMR on the copolymer and higher incorporation of glycomonomer AP hydrogels without purification or drying (Figure S2). Approximately 10 mol % of unreacted monomers remained, with no significant difference observed among the different hydrogel compositions.

ASEC-MALLS was attempted for all five hydrogel compositions in buffer solution, but molecular weight measurements could not be obtained due to limited solubility. To estimate the molecular weight of the systems, a homopolymer of GalEAm (pGal) was synthesized following the same synthetic procedure outlined in the experimental section. The model homopolymer is soluble in buffer and was determined to have *M*_n_ of 1.64 × 10^6^ g/mol, *M*_w_ of 1.92 × 10^6^ g/mol, and *Đ* of 1.17 (Figure S3), In our previous work, a homopolymer of NiPam synthesized via the same procedure was found to have *M*_w_ of 2.11 × 10^6^ g/mol by static light scattering.^32^ In other work, we showed that Gal/Glc-DMAPAm copolymers synthesized by reversible addition–fragmentation chain-transfer polymerization exhibited reaction kinetics similar to those of GalEAm and GlcEAm homopolymers.^24^ In our system, given the identical reaction conditions and comparable unreacted monomer content across formulations, we assume the co- and terpolymers will have similar high molecular weights, in the range of 1 × 10^6^ g/mol.

### Effects of Temperature

*Amblyomma americanum* is ectothermic, and host contact during hematophagy (blood-feeding) significantly influences its body temperature, which typically rises to approximately 32–35 °C. To better replicate the host interface and conditions of an ingested blood meal, we selected 37 °C as the primary experimental condition. Thermal stability of the cationic terpolymer was further assessed under elevated temperature stress, with exposure to 50 °C, a condition exceeding the known physiological tolerance range of the tick, thereby providing a stringent test of material robustness.

Increasing the temperature of the hydrogels from room temperature to 37 and 50 °C results in no change to the qualitative properties of the gel (Figure 2a-c). It has been shown that the LCST of pNiPAm homo- and copolymers is dependent on polymer stereochemistry and comonomer choice, where hydrophilic comonomers and nonisotactic stereochemistry increase the LCST.^47^ We attribute the lack of an LCST in the copolymer to the atactic stereochemistry of the polymer chain and the addition of extremely hydrophilic cationic and glyco-comonomers. It is possible that temperatures higher than 50 °C could lead to a thermal transition, but these temperatures are not relevant to the purpose of this study.

**Figure 2:**
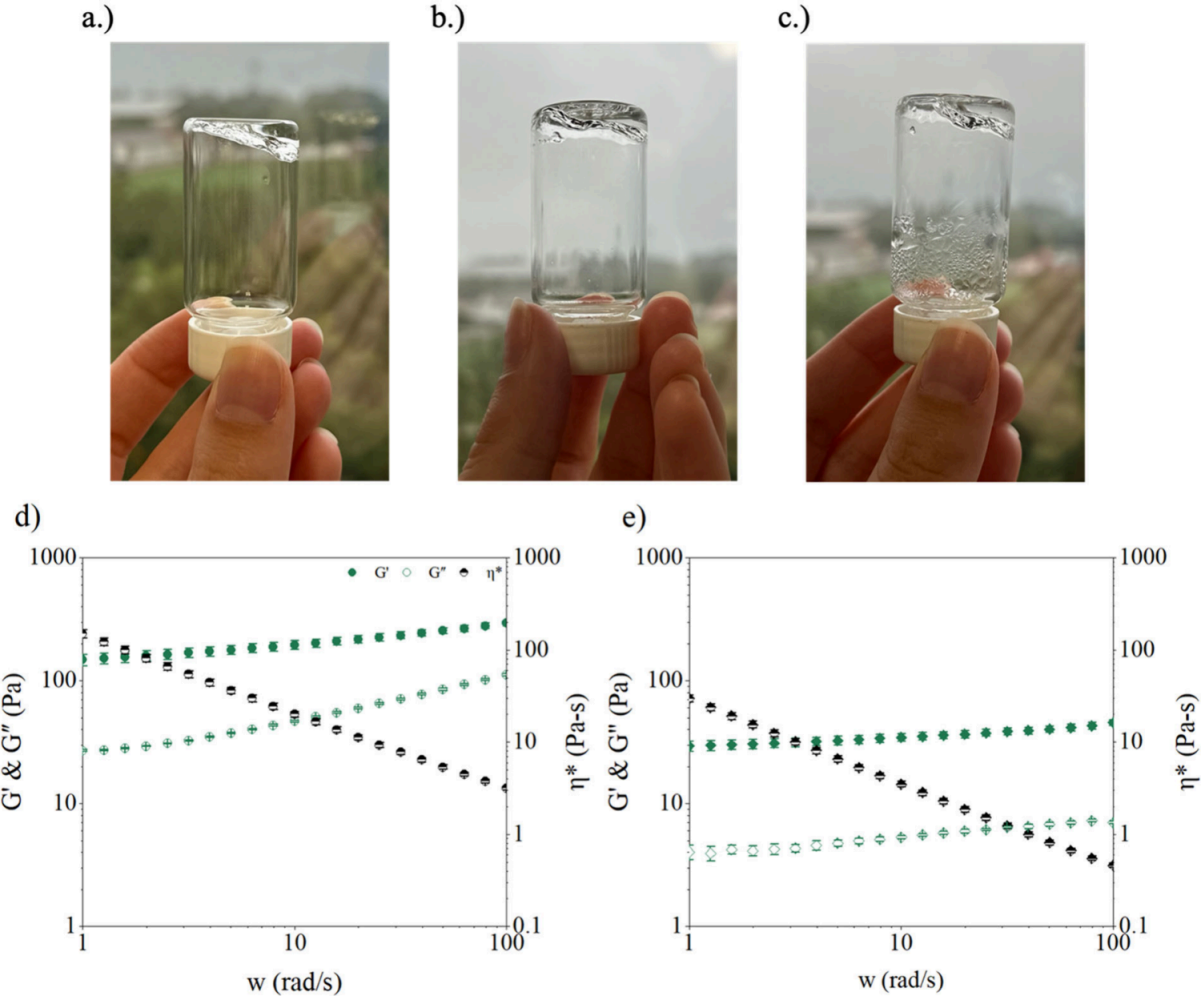
Images of pN_53_D_47_ at a) room temperature, b) 37 °C, and c) 50 °C. Frequency sweep of pGal_31_N_45_D_24_ d) as polymerized (18.6 wt % solids based on reaction formula) and e) SPure (0.38 ± 1.51, *n* = 3) wt % solids determined by TGA measurement. Rheological measurements were performed at 21 °C. Closed green symbols represent *G*′, open symbols represent *G*″, and half-filled black symbols represent η*. Data are represented as mean ± SD (*n* = 3).

### Hydrogel Rheology

#### Effects of Swelling and Composition

Frequency sweeps of pGal_31_N_45_D_24_ AP and SPure hydrogels are shown in Figure 2d,e, respectively, those of all other hydrogels are shown in Figure S4, and rheological characteristics are provided in Table S2. For all hydrogels, the storage modulus (*G*′) remains greater than the loss modulus (*G*″) across the entire frequency range, without a crossover, indicating solid-like behavior and lack of a sol–gel transition. AP hydrogels display a moderate dependence of *G*′ on frequency, as shown by the slope of *G*′, consistent with a concentrated physically cross-linked system.^48,49^ SPure hydrogels, which have undergone purification and swelling, exhibit a decrease in *G*′ and *G*″ by an order of magnitude in comparison to AP hydrogels.. This behavior agrees with the affine network theory, where *G*′ scales with polymer volume fraction and cross-link density.^50,51^ AP and SPure hydrogels exhibit shear-thinning behavior (*n* < 1), where SPure samples are shown to be more shear-thinning. Additionally, decrease in the slope of *G*′ and complex viscosity (η*) in SPure samples indicates that network quality, defined as more regular physical cross-link distribution and fewer defects, is improved with swelling.^52^

A comparative plot of *G*′ vs frequency for SPure hydrogels is shown in Figure 3a, and statistical analysis of comparative rheological properties is provided in Figure S5. In general, at low glycopolymer incorporation there is no statistically significant difference in the rheological performance of the copolymer and glycopolymer terpolymers. At the higher level of glycomonomer incorporation, on the other hand, the hydrogel strength is decreased, and network quality is reduced, evidenced by statistically significant decreases in *G*_0_′ and shear thinning index with increases in slope of *G*′, mesh size, and slope of η* in comparison to that of the copolymer. In addition, the standard deviation of the rheological properties of Spure pGlc_39_N_39_D_22_ is generally higher than that of the other hydrogels, indicating greater variability in gel strength and network quality. These differences may be attributed in part to the higher incorporation of Glc than Gal, and partly to differences in intra- and intermolecular hydrogen bonding associated with the Gal and Glc pendant saccharides, previously reported by our group.^53^ The intermolecular bonding in the Glc terpolymer leads to increased water uptake, confirmed by the statistically significant decrease in wt % solids compared to the other hydrogels, and larger mesh size and size distribution, resulting in a decrease in hydrogel strength and reduction of the network quality.

**Figure 3:**
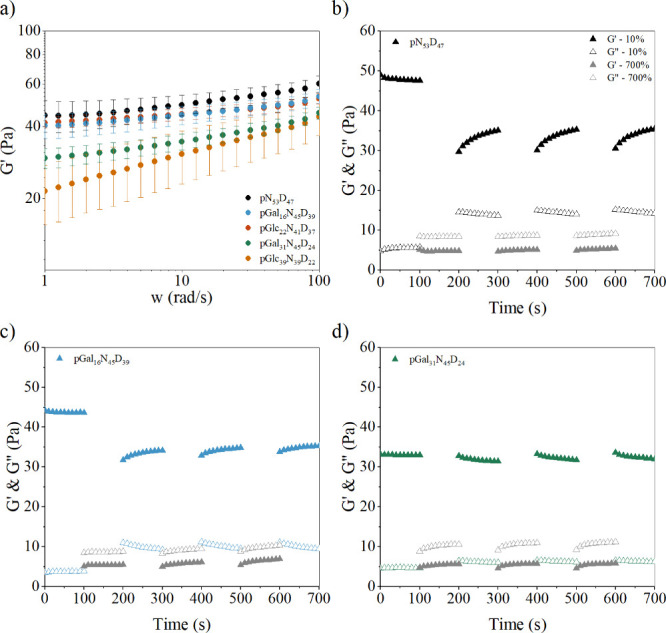
Rheological characteristics of SPure hydrogels. a) *G*′ as a function of frequency. Multicycle alternating step strain experiments of SPure_pH3 hydrogels: b) pN_53_D_47_, c) pGal_16_ *N*_45_D_39_, and d) pGal_31_N_45_D_24_. Rheological measurements were performed at 21 °C. Closed symbols represent *G*′; open symbols represent *G*″. The color corresponding to a specific hydrogel composition indicates 10% strain; gray indicates 700% strain.

Multicycle alternating step strain experiments were performed to probe the self-healing capabilities of the SPure hydrogels. Comparative plots for the copolymer with galactose terpolymers are shown in Figure 3b–d, and for glucose terpolymers in Figure S6a,b. All compositions show solid-like characteristics (*G*′ > *G*″) at 10% nondestructive strain and liquid-like characteristics (*G*″ > *G*′) at 700% destructive strain. On the return to 10% strain, the copolymer and low glyco-content terpolymers show approximately 70% recovery, from initial *G*′ of ∼45 to ∼30 Pa, and they retain the same recovered *G*′ for the remaining cycles. The high content Gal terpolymer, on the other hand, shows almost complete recovery, with initial and recovered *G*′ of ∼30 Pa maintained through all cycles (Figure 3d). The high content Glc terpolymer shows slightly lower performance with an initial *G*′ of 28 Pa and recovered *G*′ of 25 Pa (Figure S6b). It is notable that the recovered modulus is similar for all samples, with *G*′ = 30 ± 5 Pa, which remains stable through four cycles.

These results indicate that our hydrogels are well suited for spray-based applications. As proof of concept, the hydrogels were sprayed onto a vertically oriented polystyrene Petri dish using a commercially available spray bottle atomizer. The attached video (Video 1) depicts the pGal_31_N_45_D_24_ hydrogel containing a trace amount of MO for visualization. Following passage through the fine atomizer, the pGal_31_N_45_D_24_ hydrogel exhibited little to no observable flow and rapidly recovered its gelled state, consistent with the self-healing behavior observed in rheological testing. Additionally, qualitatively similar behavior was observed for the other hydrogel compositions.

#### Effects of pH Adjustment

Solutions at pH of 10 and 3 (above and well below DMAPAm p*K*_a_ of 9) were prepared to evaluate stimuli-responsive behavior of the SPure hydrogels. At pH 10, the tertiary amine of the DMAPAm is deprotonated, resulting in an increase in hydrophobicity of the chain, expulsion of water from the mesh, and aggregation of the polymer chains to break the hydrogel. In contrast, at pH 3, the DMAPAm tertiary amine is almost entirely protonated, resulting in increased hydrophilicity and chain expansion due to charge–charge repulsion. Frequency sweeps of the SPure and SPure_pH3 pGal_31_N_45_D_24_ hydrogels are shown in Figure 4a and the other hydrogels in Figure S7, with rheological characteristics given in Table S3 and statistical analysis in Figure S8. The solid-like behavior remains unchanged, but the increase in amine protonation reduces the gel quality and causes the hydrogel to become weaker and less shear-thinning. Similar trends have been observed by Gao et al.^54^ These observations are attributed to the increased charge density and charge–charge repulsion, creating an overall increase in mesh size and mesh size distribution. The pH3 hydrogels also showed increased variability and no clear differences in rheological behavior related to glycomonomer incorporation or type. This is likely due to the overwhelming effect of charge–charge repulsion at low pH, which makes any differences in saccharide group hydrogen bonding negligible.

**Figure 4:**
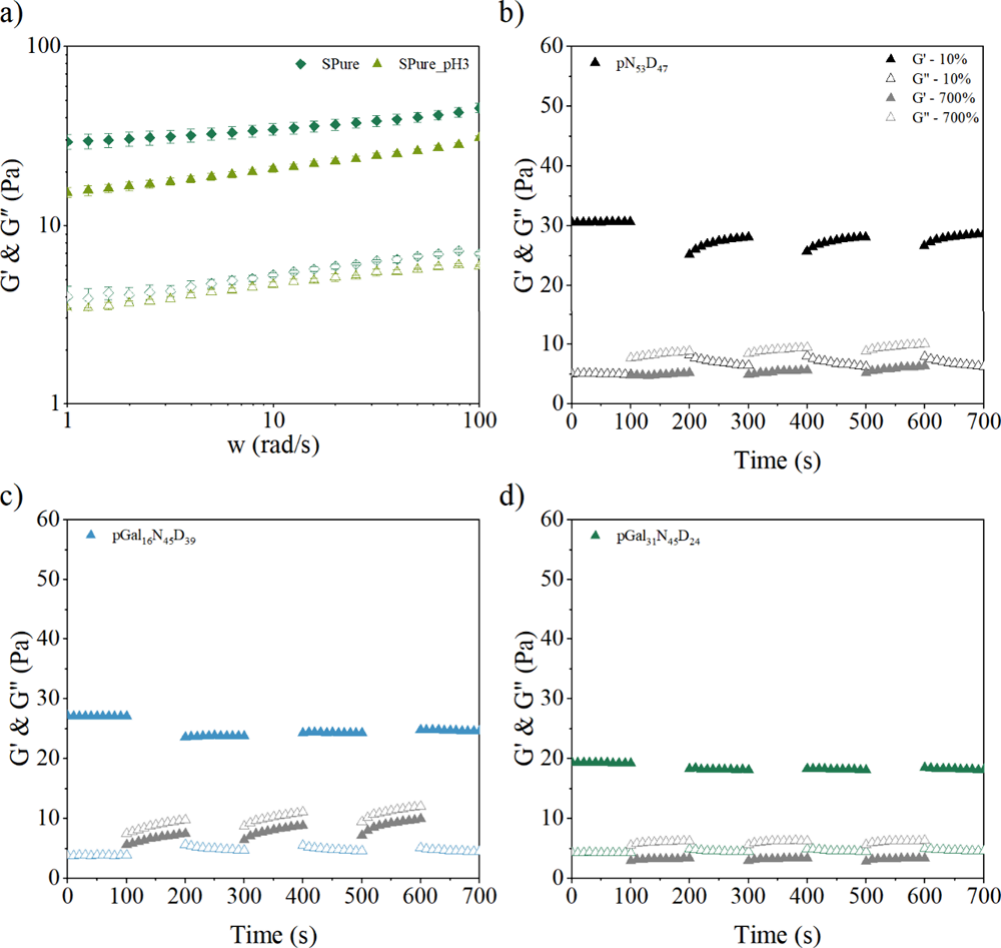
Rheological characteristics comparison of SPure and SPure_pH3 hydrogels. a) Frequency sweep of pGal_31_N_45_D_24_. Multicycle alternating step strain experiments of SPure_pH3 hydrogels: b) pN_53_D_47_, c) pGal_16_N_45_D_39_, and d) pGal_31_N_45_D_24_. Rheological measurements were performed at 21 °C. Closed symbols represent *G*′; open symbols represent *G*″. The color corresponding to a specific hydrogel composition indicates 10% strain, gray indicates 700% strain.

Multicycle alternating step strain experiments of the SPure_pH3 hydrogels show similar results to the SPure samples (Figure 4b–d and Figure S6c,d). No statistically significant difference in modulus recovery is observed for the copolymer in comparison to the low incorporation glycomonomer SPure_pH3 hydrogels. There is, however, a statistically significant improvement in modulus recovery, 88% of the initial *G*′, compared to the SPure samples. This improvement is attributed to the increased charge–charge repulsion, forcing the chains apart after removal of destructive strain. In hydrogels with increased glycomonomer loading, pH adjustment did not affect the modulus recovery, nor did glycomonomer structure.

#### Effects of dsRNA

SPure hydrogels loaded with dsRNA (N:P ratio of 25) were prepared to evaluate the effects of dsRNA strands on their rheological properties. Frequency sweeps of the SPure_RNA hydrogels (Figure S9a,c,e) show that the solid-like behavior remains unchanged. In addition, no significant differences in rheological properties are observed in any of the tested hydrogel compositions compared to the SPure hydrogels. Similar results are observed in multicycle alternating step strain experiments, where the recovery behavior shows no significant differences (Figure S9b,d,f). Rheological properties may be affected at lower N:P ratios, where higher RNA loading and stronger electrostatic interactions would be more likely to influence bulk rheology.

### Release Studies of Methyl Orange

A small molecule anionic dye, MO, was selected for evaluation of release of a negatively charged load from the cationic hydrogels. We recognize that MO does not adequately model the size and interactions of RNA, and these results are presented for characterization of hydrogel behavior and to aid in the down selection of hydrogels for in vivo and in vitro RNA studies. For the SPure hydrogels (Figure 5a and Table S4), all compositions show an initial high rate of release, followed by a slower release phase. During the first 60 h, no significant difference in cumulative MO release (14%) is observed between the copolymer and the low incorporation of glycomonomer hydrogels. In contrast, hydrogels with higher incorporation of glycomonomer exhibit a statistically significant increase in cumulative release compared to the copolymer, consistent with the lower cationic content of these hydrogels. Cumulative release appears to plateau after 60 h, with higher levels of release for the hydrogels with high glycomonomer and low cationic content. The mesh size may also play a role in the rate of diffusion through the hydrogel,^55^ as the high glycomonomer content hydrogels have larger mesh size (Table S2). Modeling release in similar systems where cargo-matrix binding is significant has become a topic of interest, particularly with the rise of pH-responsive materials. It is challenging to model such systems due to their multiple modes of release with additional individual kinetic profiles beyond simple Fickian transport.^56,57^ Trends in cumulative release of MO in the SPure_pH3 samples (Figure 5b, Table S4) are similar to those observed for SPure samples, with the high glycomonomer-loaded hydrogels with the lowest cationic content showing higher cumulative release than the other systems. In general, initial release rates are somewhat lower at pH3, where greater protonation of the amine results in higher interaction with the anionic cargo.

**Figure 5:**
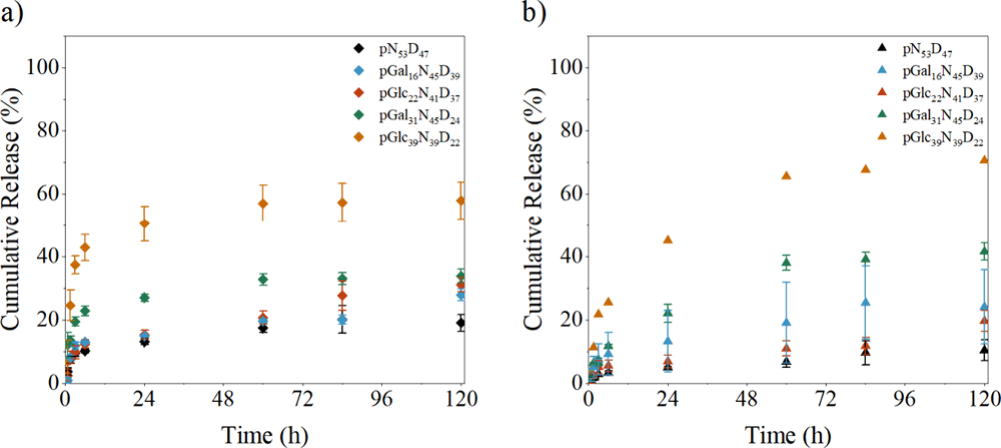
Cumulative release of MO from a) SPure and b) SPure_pH3 hydrogels. Data are represented as mean ± SD (*n* = 3).

Based on the MO release data, we chose to pursue RNA studies with the copolymer and high glycomonomer content terpolymer hydrogels, which show the greatest differences in MO release and rheological performance.

### Preliminary In Vivo Study

A preliminary in vivo study was conducted to evaluate hydrogel-mediated uptake in the tick digestive tract, defined here as transport across the peritrophic membrane into the midgut. Copolymers comprising a galactose-based monomer with either NiPAm or DMAPAm were selected to assess the effects of saccharide-functional polymers with and without a cationic comonomer. A galactose-containing terpolymer was included to probe potential synergistic effects arising from the combination of all three monomers. Among the three hydrogel formulations evaluated (pGal_41_N_59_, pGal_56_D_44_, and pGal_25_N_34_D_41_), only the cationic terpolymer hydrogel successfully traversed the peritrophic membrane (Figure 6a,b). While this result suggests a synergistic effect of the three monomer functionalities, the underlying transport mechanism requires further investigation

**Figure 6:**
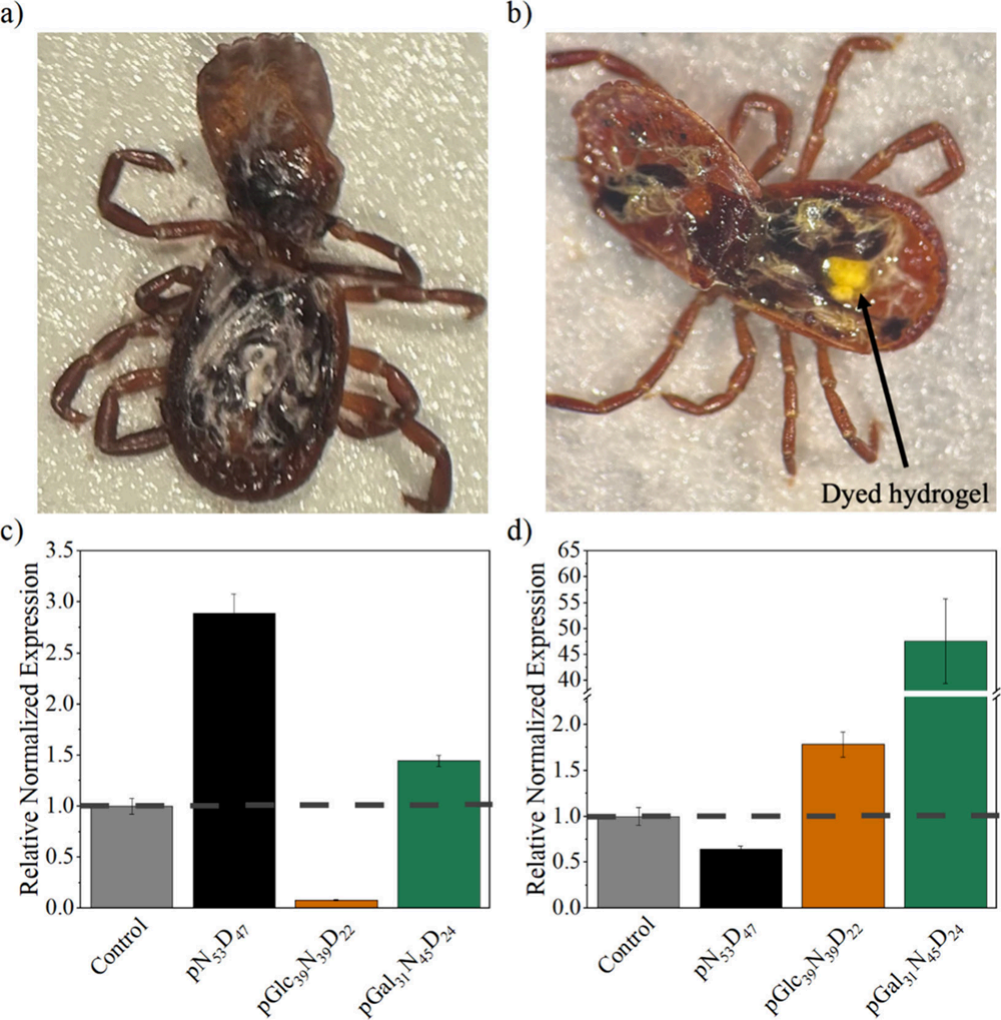
Images of dissected ticks showing a) no visible dyed pGal_41_N_59_ hydrogel and b) visible accumulation of dyed pGal_25_N_34_D_41_ hydrogel in the distal tissue. Knockdown of Galectin alters gene expression in partially blood-fed tick tissues: c) ovaries and d) midgut. Data are represented as mean ± SEM. Expression levels were normalized to the reference gene Aa β-actin.

### Galectin Silencing (RNA Interference) Proof-of-Concept In Vivo Study

Delivering dsRNA in combination with the glycopolymers to target the Galectin gene in the Lone Star tick disrupts important physiological and biochemical processes: The midgut is the main site for blood meal digestion, nutrient absorption, and interaction with the tick microbiome. Knocking down Galectin in the midgut reduces galactose-metabolism-related gene expression. This hampers the tick’s ability to process the blood meal, impairs digestion, and affects microbial maintenance. Meanwhile, the salivary glands produce saliva containing proteins that act with anti-inflammatory, anesthetic, and immunomodulatory properties. Suppressing Galectin in the salivary glands lowers the tick’s ability to modulate the host’s immune response and changes the expression of Galectin. The ovaries are responsible for oogenesis and embryogenesis, directly affecting the tick’s high reproductive capacity (fecundity). Delivering dsRNA to the ovaries inhibits genes necessary for egg development, resulting in impaired oviposition, fewer viable eggs, and decreased population growth.

To assess the role of hydrogels in facilitating dsRNA delivery to the midgut, salivary gland, and ovarian tissues, RNAi-mediated knockdown experiments targeting tick galectin were performed (Figure 6c,d and Figure S10). The copolymer hydrogel facilitated gene knockdown in the midgut, whereas the glucose-based cationic terpolymer hydrogels enabled gene knockdown in ovarian tissue (Figure 6c,d). In contrast, none of the dsRNA-conjugated hydrogel formulations induced silencing of galectin expression in the salivary glands (Figure S10).

These results are attributed to differences in hydrogel composition and transport behavior. In the preliminary in vivo study, the galactose-based cationic terpolymer (pGal_25_N_34_D_41_) was shown to traverse the peritrophic membrane, enabling delivery to distal tissues. Based on the shared cationic terpolymer architecture, we infer that the glucose-based terpolymer may exhibit similar transport capability, although this has not been directly demonstrated. Notably, the glucose-based terpolymer produced the largest reduction in galectin expression in ovarian tissue, suggesting effective delivery to this distal site. In contrast, the nonsaccharide copolymer hydrogel appears unable to cross the peritrophic membrane and remains confined to the midgut, where localized dsRNA release results in effective gene silencing. This behavior is consistent with the preliminary transport studies in which the other copolymer compositions (pGal_41_N_59_ and pGal_56_D_44_) were unable to traverse the membrane, supporting the hypothesis that synergistic interactions among the three monomer functionalities in the terpolymer are required for membrane transport.

Although we were able to show that the target gene’s mRNA transcript levels had significantly decreased in our trials, we were unable to find any appreciable changes in survival, feeding behavior, or reproductive output during the study. We postulate multiple explanations for this silent knockdown: Because the target protein may have a lengthy half-life, existing protein stockpiles were nevertheless adequate to sustain biological function during the observation period despite lower mRNA levels. The effects of the particular gene silencing may have been lessened by paralogous genes or possible compensatory processes. It is possible that the level of silencing attained was below the essential threshold needed to cause a macroscopic phenotypic change.

In our previous work, we observed that galactose-based polyelectrolyte complexes (PECs) enhanced uptake in tick cell lines. However, the transition to an *in vivo* model using cationic terpolymer hydrogels introduces additional physiological barriers, particularly the midgut and hemolymph, which are absent in the cell culture system.

We propose a multifaceted hypothesis to explain why the glucose-based hydrogel demonstrated superior delivery in a technically challenging tissue (ovaries) to deplete gene expression, compared to the galactose-based formulation. The midgut of a feeding tick is a highly metabolically active and selective interface. We hypothesize that the galactose-based terpolymers may exhibit *high* affinity for galactose-binding lectins within the midgut epithelium or associated with the peritrophic matrix, leading to sequestration or retention within gut tissues. This “trapped” effect could limit systemic dissemination and reduce delivery to distal organs such as ovaries. In contrast, the glucose-based terpolymer may engage in fewer specific lectin-mediated interactions, enabling more efficient passage into the hemolymph and improved distribution.

In addition, galectin expression is known to be responsive to ligand availability. It is possible that the galactose-containing hydrogel, acting as a high-density ligand source, induces a compensatory upregulation of galectins in ovarian tissue as a physiological response to the delivery system itself. High local concentrations of galactose moieties may also compete with endogenous galectin-glycan interactions, potentially triggering feedback regulation aimed at restoring functional binding capacity.

Our results demonstrate a tiered efficiency in tissue-specific delivery that depends on polymer composition. The cationic copolymer functioned effectively as a delivery vehicle for dsRNA delivery in the midgut, achieving robust local gene silencing. However, systemic delivery to distal tissues required the added structural and functional complexity of the glucose-based cationic terpolymer, which was the only formulation that produced significant gene knockdown in ovarian tissue.

Successful delivery to the ovaries represents an important advance in tick functional genomics, as this organ is enclosed by the multiple basal laminae and has historically been considered a “difficult-to-reach” tissue for noninjected RNAi approaches.^16^ The ability of the glucose-based terpolymer to preserve cargo integrity during hemolymph transit and support ovarian uptake suggests a potential role for the glucose glycomonomer in enhancing systemic stability, transport efficiency, or barrier penetration.

## Limitations and Future Directions

We explicitly acknowledge that the absence of protein-level validation and phenotypic measurements limits interpretation of the biological outcomes. Although successful silencing of target genes in distal tissues is consistent with traversal of the peritrophic matrix, the underlying biophysical mechanism remains unresolved. Whether transport occurs via paracellular pathways, transient pore formation, or active transcytosis is still an open question and requires further investigation. To address this, we propose future studies employing approaches such as gold-nanoparticles labeling or FRET-based sensors to monitor hydrogel integrity and track its movement in real time. Finally, while the biochemical properties of the terpolymer indicate potential broad applicability, systemic validation across multiple species and a diverse set of genetic targets will be necessary to establish its generalizability and translational relevance.

## Conclusion

In this work, we developed novel cationic-glycopolymer hydrogels with reversible gelation and pH responsiveness. Glycomonomer structure and cationic incorporation were found to be key drivers of material behavior, influencing network structure, gel strength, and cargo transport. Small molecule cargo release was tunable through both the type and amount of glycomonomer, indicating the ability to control delivery performance through monomer selection. As a proof of concept, we show that hydrogel traversal of the peritrophic membrane and subsequent gene silencing in distal tissue requires incorporation of both DMAPAm and NiPAm monomers in the hydrogel network and is associated with the specific structure of the glycomonomer, which generates a distinct terpolymer architecture. In contrast, simpler copolymer systems of glycomonomer with either DMAPAm or NiPAm remain largely restricted to the midgut. These results suggest that distal tissue penetration is not an inherent property of terpolymer systems but is instead linked to specific physiochemical features conferred by the DMAPAm and glycomonomer subunits, including tuned charge density and enhanced hydrogen bonding capacity within the hydrogel matrix. Together, these findings establish a design framework for tailoring glycopolymer hydrogel-based delivery systems with the potential of sprayable application.

## Supplementary Material





## Notes

Primer sequences used for galectin silencing and gene expression analysis; representative ^1^H NMR spectra of copolymer and terpolymer hydrogels and unpurified samples; ASEC-MALLS trace for the GalEAm homopolymer (pGal); rheological properties, mesh size calculations, and weight percent solids of AP, SPure, and pH-adjusted hydrogels; frequency sweep and multicycle step-strain rheological data of AP, SPure, and pH-adjusted hydrogels; statistical analysis of rheological data; multicycle alternating step strain experiments; rheological properties; storage modulus comparison for hydrogels of different pH; rheological characterization of dsRNA-loaded hydrogels; cumulative methyl orange release profiles under neutral and acidic conditions; gene expression data from in vivo RNA interference experiments (PDF)
Video of spray behavior (MOV)

## References

[1] Crispell G., Commins S. P., Archer-Hartman S. A., Choudhary S., Dharmarajan G., Azadi P., Karim S. (2019). Discovery of alpha-gal-containing
antigens in North American tick species believed to induce red meat
allergy. Frontiers in immunology.

[2] Sharma S. R., Karim S. (2021). Tick saliva and the
alpha-gal syndrome: finding a needle in a haystack. Frontiers in cellular and infection microbiology.

[3] Ross M., Daley G., Burnard M. L., Sen A., Beyene E., Bisrat M., Zinabu S. W., Abrie M., Michael M. (2025). Alpha-Gal
on the Rise: The Alarming Growth of Alpha-Gal Syndrome in High-Risk
Regions. Cureus.

[4] Paddock C. D., Childs J. E. (2003). Ehrlichia chaffeensis:
a prototypical emerging pathogen. Clin. Microbiol.
Rev..

[5] Varela A. S., Luttrell M. P., Howerth E. W., Moore V. A., Davidson W. R., Stallknecht D. E., Little S. E. (2004). First culture isolation
of Borrelia
lonestari, putative agent of southern tick-associated rash illness. J. Clin. Microbiol..

[6] Kugeler K. J., Schwartz A. M., Delorey M. J., Mead P. S., Hinckley A. F. (2021). Estimating
the Frequency of Lyme Disease Diagnoses, United States, 2010–2018. Emerg Infect Dis.

[7] Hojgaard A., Eisen R. J., Piesman J. (2008). Transmission
dynamics of Borrelia
burgdorferi ss during the key third day of feeding by nymphal Ixodes
scapularis (Acari: Ixodidae). Journal of medical
entomology.

[8] des
Vignes F., Piesman J., Heffernan R., Schulze T. L., Stafford K. C., Fish D. (2001). Effect of
tick removal on transmission of Borrelia burgdorferi and Ehrlichia
phagocytophila by Ixodes scapularis nymphs. Journal of infectious diseases.

[9] Barnard A.-C., Nijhof A. M., Fick W., Stutzer C., Maritz-Olivier C. (2012). RNAi in arthropods:
insight into the machinery and applications for understanding the
pathogen-vector interface. Genes.

[10] Velez A. M., Fishilevich E. (2018). The mysteries
of insect RNAi: A focus on dsRNA uptake
and transport. Pestic. Biochem. Physiol..

[11] Galay, R. L. ; Umemiya-Shirafuji, R. ; Mochizuki, M. ; Fujisaki, K. ; Tanaka, T. RNA interference - a powerful functional analysis tool for studying tick biology and its control. RNA Intererence; InTech: 2016; p 411–445, 10.5772/61577.

[12] Adamson S. W., Browning R. E., Budachetri K., Ribeiro J. M., Karim S. (2013). Knockdown
of selenocysteine-specific elongation factor in Amblyomma maculatum
alters the pathogen burden of Rickettsia parkeri with epigenetic control
by the Sin3 histone deacetylase corepressor complex. PLoS One.

[13] Villarreal A. M., Adamson S. W., Browning R. E., Budachetri K., Sajid M. S., Karim S. (2013). Molecular characterization and functional
significance of the Vti family of SNARE proteins in tick salivary
glands. Insect biochemistry and molecular biology.

[14] Browning R., Karim S. (2013). RNA interference-mediated depletion of N-ethylmaleimide Sensitive
Fusion Protein and Synaptosomal Associated Protein of 25 kDa results
in the inhibition of blood feeding of the G ulf C oast tick, A mblyomma
maculatum. Insect Molecular Biology.

[15] Karim S., Kenny B., Troiano E., Mather T. N. (2008). RNAi-mediated gene
silencing in tick synganglia: a proof of concept study. BMC biotechnology.

[16] Karim, S. ; Adamson, S. W. RNA interference in ticks: a functional genomics tool for the study of physiology. Advances in insect physiology; Elsevier: 2012; Vol. 42, pp 119–154.

[17] Jiang, X. ; Abedi, K. ; Shi, J. Polymeric nanoparticles for RNA delivery. In Encyclopedia of Nanomaterials, First ed.; Yin, Y. , Lu, Y. , Xia, Y. , Eds.; Elsevier: Oxford, 2023; pp 555–573.

[18] Quilez-Molina A. I., Niño Sanchez J., Merino D. (2024). The role of polymers in enabling
RNAi-based technology for sustainable pest management. Nat. Commun..

[19] Cai X., Dou R., Guo C., Tang J., Li X., Chen J., Zhang J. (2023). Cationic polymers as transfection
reagents for nucleic acid delivery. Pharmaceutics.

[20] Loginova T. P., Khotina I. A., Kabachii Y. A., Kochev S. Y., Abramov V. M., Khlebnikov V. S., Kulikova N. L., Mezhuev Y. O. (2023). Promising gene delivery
properties of polycations based on 2-(N, N-dimethylamino) ethyl methacrylate
and polyethylene glycol monomethyl ether methacrylate copolymers. Polymers.

[21] Parsons K. H., Mondal M. H., McCormick C. L., Flynt A. S. (2018). Guanidinium-Functionalized
Interpolyelectrolyte Complexes Enabling RNAi in Resistant Insect Pests. Biomacromolecules.

[22] Van
Bruggen C., Hexum J. K., Tan Z., Dalal R. J., Reineke T. M. (2019). Nonviral Gene Delivery with Cationic Glycopolymers. Acc. Chem. Res..

[23] Tortajada L., Felip-León C., Vicent M. J. (2022). Polymer-based non-viral vectors for
gene therapy in the skin. Polym. Chem..

[24] Stockmal K.
A., Downs L. P., Davis A. N., Kemp L. K., Karim S., Morgan S. E. (2022). Cationic
Glycopolyelectrolytes for RNA Interference
in Tick Cells. Biomacromolecules.

[25] Yu N., Christiaens O., Liu J., Niu J., Cappelle K., Caccia S., Huvenne H., Smagghe G. (2013). Delivery of dsRNA for
RNAi in insects: an overview and future directions. Insect Science.

[26] Shoukat H., Buksh K., Noreen S., Pervaiz F., Maqbool I. (2021). Hydrogels
as potential drug-delivery systems: Network design and applications. Therapeutic Delivery.

[27] Wei Z., Yang J. H., Zhou J., Xu F., Zrínyi M., Dussault P. H., Osada Y., Chen Y. M. (2014). Self-healing gels
based on constitutional dynamic chemistry and their potential applications. Chem. Soc. Rev..

[28] Talebian S., Mehrali M., Taebnia N., Pennisi C. P., Kadumudi F. B., Foroughi J., Hasany M., Nikkhah M., Akbari M., Orive G. (2019). Self-healing hydrogels:
The next paradigm shift in tissue engineering?. Advanced Science.

[29] Wang S., Zheng H., Zhou L., Cheng F., Liu Z., Zhang H., Wang L., Zhang Q. (2020). Nanoenzyme-Reinforced
Injectable Hydrogel for Healing Diabetic Wounds Infected with Multidrug
Resistant Bacteria. Nano Lett..

[30] Xu D., Hawk J. L., Loveless D. M., Jeon S. L., Craig S. L. (2010). Mechanism
of shear thickening in reversibly cross-linked supramolecular polymer
networks. Macromolecules.

[31] Marco-Dufort B., Iten R., Tibbitt M. W. (2020). Linking
molecular behavior to macroscopic
properties in ideal dynamic covalent networks. J. Am. Chem. Soc..

[32] Wang X., Abernathy H. G., Kemp L. K., Morgan S. E. (2024). Sprayable adhesive
glycopolymer hydrogels with rapid in-situ gelation. Polym. Chem..

[33] Gheysoori P., Paydayesh A., Jafari M., Peidayesh H. (2023). Thermoresponsive
nanocomposite hydrogels based on Gelatin/poly (N-isopropylacrylamide)­(PNIPAM)
for controlled drug delivery. Eur. Polym. J..

[34] Throat S., Bhattacharya S. (2024). Macromolecular
Poly (N-isopropylacrylamide)­(PNIPAM)
in Cancer Treatment and Beyond: Applications in Drug Delivery, Photothermal
Therapy, Gene Delivery and Biomedical Imaging. Adv. Polym. Technol..

[35] Boyaci T., Orakdogen N. (2015). pH-responsive poly (N, N-dimethylaminoethyl methacrylate-co-2-acrylamido-2-methyl-propanosulfonic
acid) cryogels: Swelling, elasticity and diffusive properties. RSC Adv..

[36] Das P. K., Dean D. N., Fogel A. L., Liu F., Abel B. A., McCormick C. L., Kharlampieva E., Rangachari V., Morgan S. E. (2017). Aqueous RAFT Synthesis of Glycopolymers
for Determination
of Saccharide Structure and Concentration Effects on Amyloid β
Aggregation. Biomacromolecules.

[37] Moncure P. J., Simon Z. C., Millstone J. E., Laaser J. E. (2022). Relationship between
gel mesh and particle size in determining nanoparticle diffusion in
hydrogel nanocomposites. J. Phys. Chem. B.

[38] Tsuji Y., Li X., Shibayama M. (2018). Evaluation of mesh size in model polymer networks consisting
of tetra-arm and linear poly (ethylene glycol) s. Gels.

[39] Zambelli A. M., Brothers K. M., Hunt K. M., Romanowski E. G., Nau A. C., Dhaliwal D. K., Shanks R. M. (2015). Diffusion
of antimicrobials
across silicone hydrogel contact lenses. Eye
& contact lens.

[40] Patrick C. D., Hair J. A. (1975). Laboratory Rearing Procedures and
Equipment for Multi.
Host Ticks (Acarina: Ixodidae). Journal of medical
entomology.

[41] Karim S., Singh P., Ribeiro J. M. (2011). A deep insight into
the sialotranscriptome
of the gulf coast tick, Amblyomma maculatum. PloS one.

[42] Adegoke A., Hanson J., Smith R. C., Karim S. (2024). Ehrlichia
chaffeensis
co-opts phagocytic hemocytes for systemic dissemination in the Lone
Star tick, Amblyomma americanum. Journal of
Innate Immunity.

[43] Karim S., Browning R., Ali L., Truhett R. (2012). Laboratory-infected
Ehrlichia chaffeensis female adult Amblyomma americanum salivary glands
reveal differential gene expression. Journal
of medical entomology.

[44] Bullard R. L., Williams J., Karim S. (2016). Temporal gene
expression analysis
and RNA silencing of single and multiple members of gene family in
the lone star tick Amblyomma americanum. PLoS
One.

[45] Green K. A., Kulkarni A. S., Jankoski P. E., Worden R. M., Loving B. M., Derbigny B., Clemons T. D., Watkins D. L., Morgan S. E. (2025). Influence
of the Saccharide Structure on Cargo Loading, Thermal Properties,
and Lectin Binding of Amphiphilic Glycopolymer-Polylactic Acid Block
Copolymer Nanoparticles. Bioconjugate chemistry..

[46] Von der Ehe, C. ; Kretschmer, F. ; Weber, C. ; Crotty, S. ; Stumpf, S. ; Hoeppener, S. ; Gottschaldt, M. ; Schubert, U. RAFT Copolymerization of Thioglycosidic Glycomonomers with N IPAm and Subsequent Immobilization onto Gold Nanoparticles. Controlled Radical Polymerization: Materials; American Chemical Society: 2015; pp 221–256.

[47] de
Oliveira T. E., Mukherji D., Kremer K., Netz P. A. (2017). Effects
of stereochemistry and copolymerization on the LCST of PNIPAm. J. Chem. Phys..

[48] Yu A. C., Smith A. A. A., Appel E. A. (2020). Structural considerations for physical
hydrogels based on polymer-nanoparticle interactions. Molecular Systems Design & Engineering.

[49] Derkach S., Zhabyko I., Voron’ko N., Maklakova A., Dyakina T. (2015). Stability and the rheological properties
of concentrated
emulsions containing gelatin-κ-carrageenan polyelectrolyte complexes. Colloids Surf., A.

[50] Tanaka, F. Polymer Physics: Applications to Molecular Association and Thermoreversible Gelation; Cambridge University Press: Cambridge, 2011.

[51] Song G., Zhao Z., Peng X., He C., Weiss R., Wang H. (2016). Rheological behavior of tough PVP-in
situ-PAAm hydrogels physically
cross-linked by cooperative hydrogen bonding. Macromolecules.

[52] Rubinstein, M. ; Colby, R. H. Polymer Physics; Oxford University Press: 2003.

[53] Bristol A. N., Saha J., George H. E., Das P. K., Kemp L. K., Jarrett W. L., Rangachari V., Morgan S. E. (2020). Effects of Stereochemistry
and Hydrogen Bonding on Glycopolymer-Amyloid-β Interactions. Biomacromolecules.

[54] Gao G., Xu W. Z., Kadla J. F. (2015). Reversible
pH-responsive hydrogels
of softwood kraft lignin and poly [(2-dimethylamino) ethyl methacrylate]-based
polymers. J. Wood Chem. Technol..

[55] Amsden B. G. (2022). Hydrogel
Mesh Size and Its Impact on Predictions of Mathematical Models of
the Solute Diffusion Coefficient. Macromolecules.

[56] Carr E. J. (2024). Total fraction
of drug released from diffusion-controlled delivery systems with binding
reactions. Int. J. Heat Mass Transfer.

[57] Pontrelli G., Toniolo G., McGinty S., Peri D., Succi S., Chatgilialoglu C. (2021). Mathematical
modelling of drug delivery from pH-responsive
nanocontainers. Computers in Biology and Medicine.

