# Supplementary material for: Reversible Cationic Glycopolymer Hydrogels for RNA Delivery in Ticks
Source: ACS Polym Au. 2026 Jun 14;6(4):1200–10. doi: 10.1021/acspolymersau.6c00064 (PMC13479872; doi:10.1021/acspolymersau.6c00064)
Supplement: Supplementary file 1 [file lg6c00064_si_001.pdf]

Supporting Information

Reversible Cationic Glycopolymer Hydrogels for RNA Delivery in Ticks

Zoe A.B. Lequeux[1], Kundu Thapa[1], Musa Rabiu[2], Alec N. Simmons[1], Laila C.W. Peery[1], John F. Searles[1], Shahid Karim[2], Sarah E. Morgan[1*]

[1]School of Polymer Science and Engineering, University of Southern Mississippi, 118 College Drive # 5050, Hattiesburg, MS 39406

[2]School of Biological, Environmental, and Earth Sciences, University of Southern Mississippi, 118 College Drive #5018, Hattiesburg, MS 39406

*Corresponding Author

Table S1. *List of primers used for gene expression.*

Primer	Forward	Reverse	Accession	Size (bp)
T7-Aa-Cystatin	5′-TAA TAC GAC TCA CTA TAG GGG CGA CAG AGA GTT CTT CGA CAC -3′	5′- TAA TAC GAC TCA CTA TAG GGG TTC AGC CAA GGA ACA TCA TAG -3′).	EU046644	246
Aa β-Actin	TGGTATCCTCACCC TGAAGTA ACGCAGCTCGTTGT AGAAG	TGGTATCCTCACCCT GAAGTA ACGCAGCTCGTTGTA GAAG	EZ000248.1	100
qPCR-Aam-37720 Galectin	AACGGGCACTACT ACCTACA	AAGATGCCAGCAGC ACAT	GBZX0100 1984.1	93
T7-Aam-Galectin	GAATAATACGACT CACTATAGGGAAC GGGCACTACTACCT ACA	GAATAATACGACTCA CTATAGGGAAGATG CCAGCAGCACAT	GBZX0100 1984.1	139

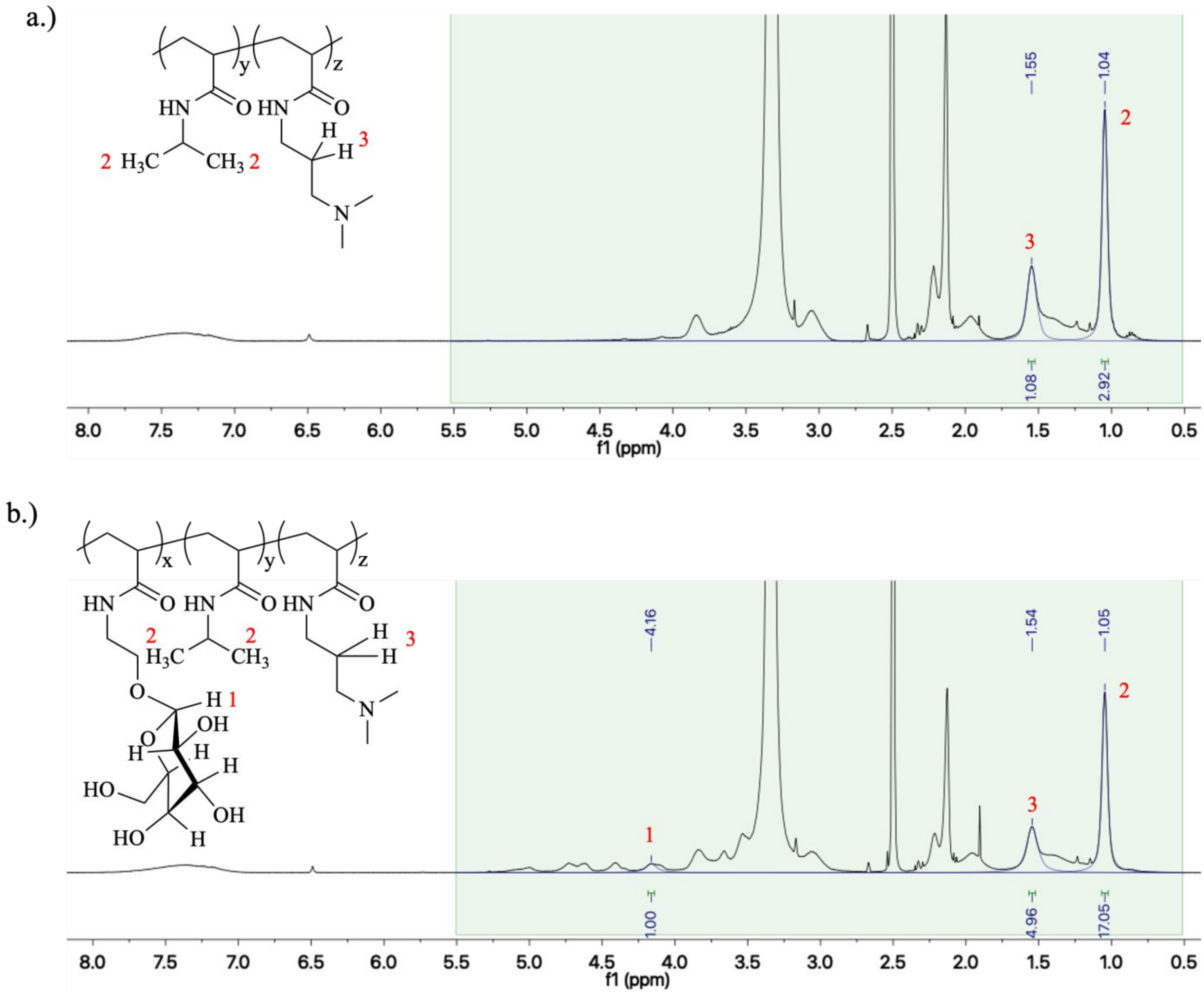

Figure S1. *^{1}H NMR of a.) $pN_{53}D_{47}$ and b.) $pGal_{31}N_{45}D_{24}$ in DMSO-d_6.*

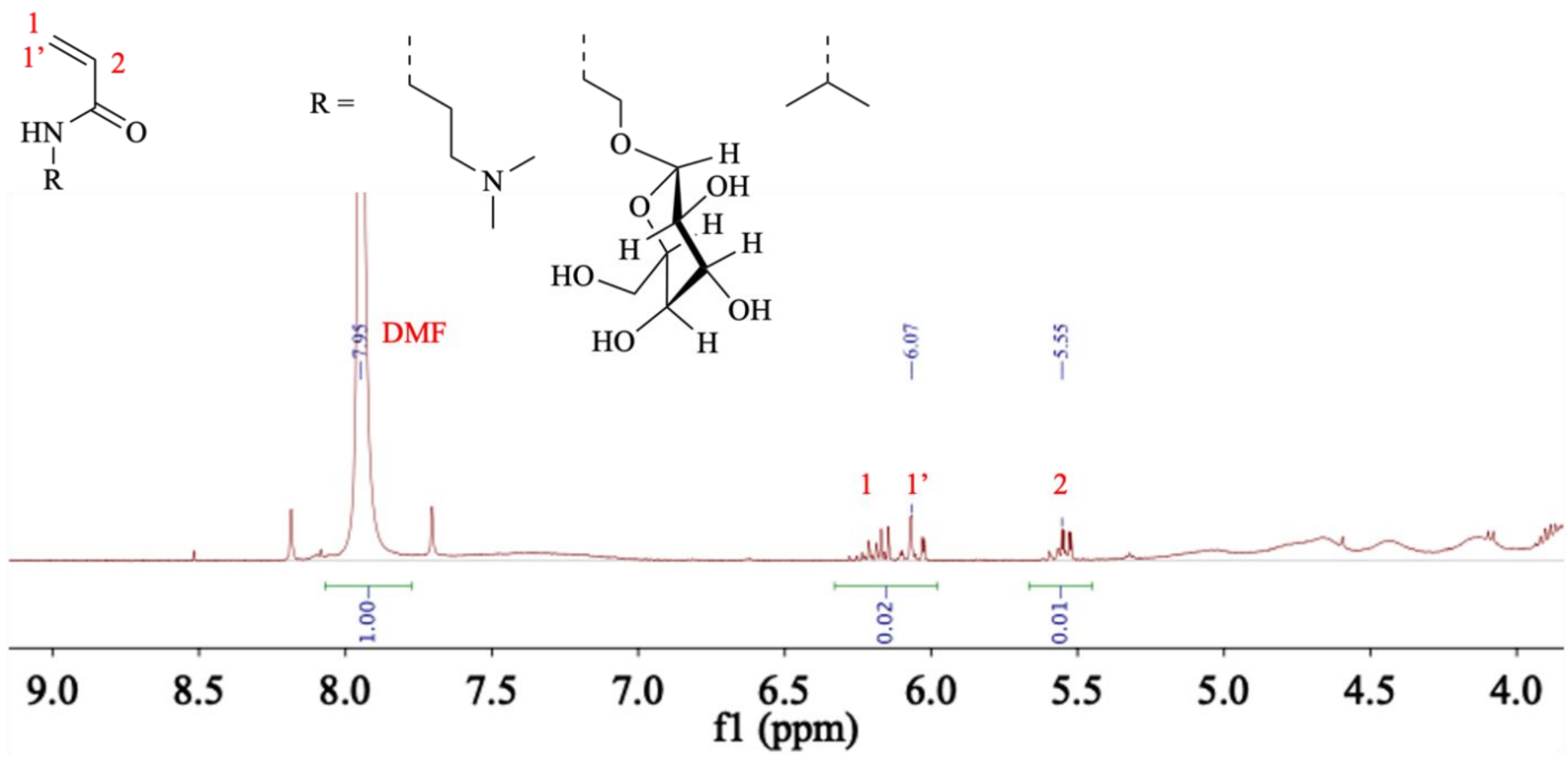

Figure S2. *^{1}H NMR of pGal$_{31}$N$_{45}$D$_{24}$ in DMSO-d$_6$ with no purification or drying.*

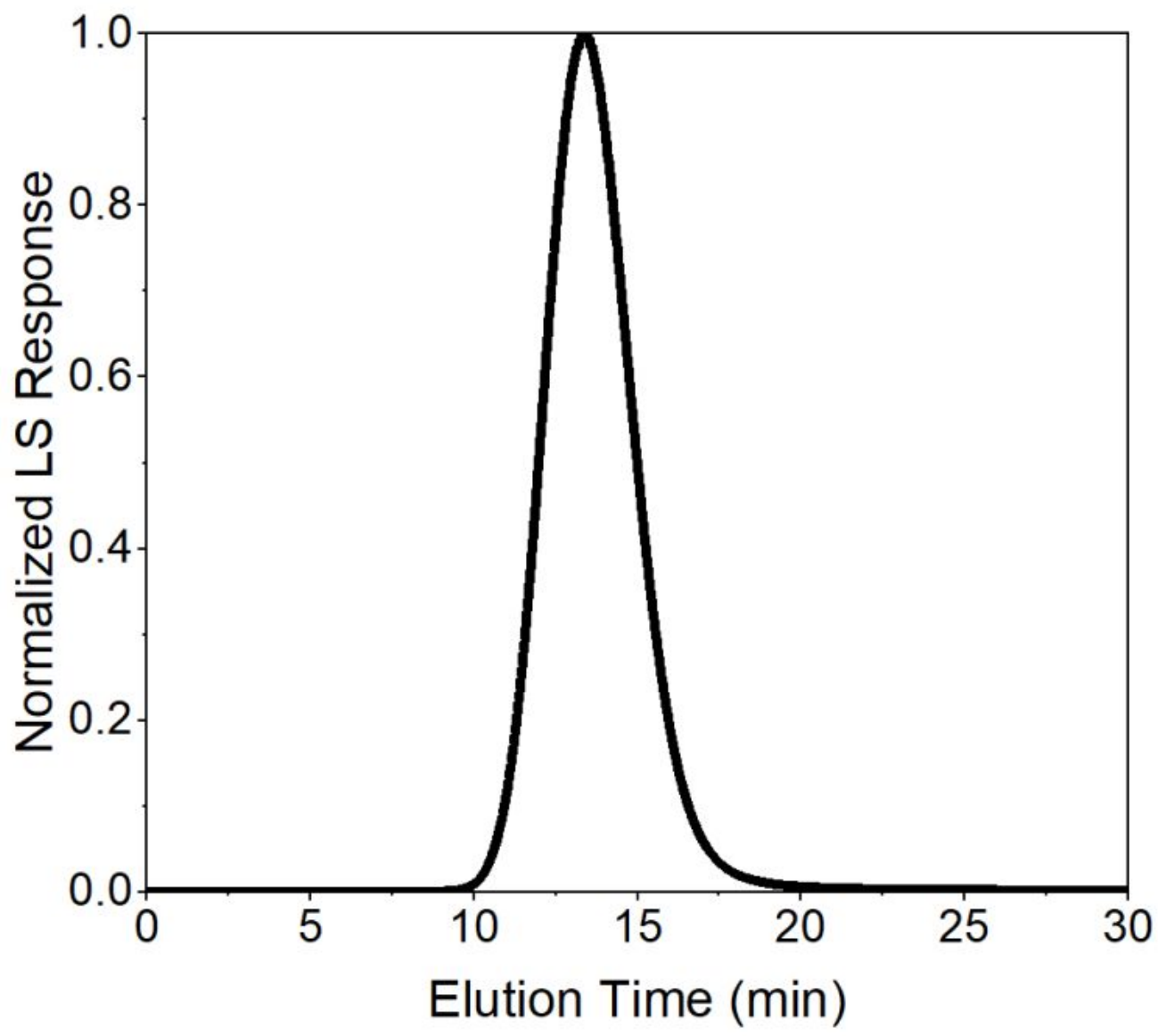

Figure S3. *ASEC-MALLS trace of pGal in buffer solution.*

Table S2. *Rheological properties and wt% solids of AP hydrogels and SPure hydrogels. Rheological measurements were performed at 21 °C.*

Hydrogel	AP / Swollen	G_0' (Pa)	Mesh size (nm)	Slope of G'	Shear-thinning index	Slope of η*	Wt% Solids
$pN_{53}D_{47}$	AP	91 ±10	36 ±1	0.20 ±0.02	0.22 ±0.02	-0.78 ±0.02	-
	Swollen	47 ±1	44 ±0	0.05 ±0.01	0.05 ±0.01	-0.95 ±0.01	0.32
$pGal_{16}N_{45}D_{39}$	AP	97 ±13	35 ±2	0.18 ±0.02	0.20 ±0.02	-0.80 ±0.02	-
	Swollen	39 ±5	47 ±2	0.06 ±0.02	0.06 ±0.02	-0.94 ±0.02	0.35
$pGlc_{22}N_{41}D_{37}$	AP	61 ±2	40 ±0	0.22 ±0.00	0.24 ±0.00	-0.76 ±0.00	-
	Swollen	41 ±2	46 ±1	0.04 ±0.00	0.05 ±0.00	-0.96 ±0.00	0.36
$pGal_{31}N_{45}D_{24}$	AP	143 ±15	31 ±1	0.15 ±0.01	0.16 ±0.01	-0.84 ±0.01	-
	Swollen	29 ±3	52 ±2	0.09 ±0.01	0.09 ±0.01	-0.91 ±0.01	0.38
$pGlc_{39}N_{39}D_{22}$	AP	125 ±11	32 ±1	0.18 ±0.01	0.19 ±0.01	-0.81 ±0.01	-
	Swollen	22 ±7	58 ±5	0.16 ±0.02	0.15 ±0.02	-0.85 ±0.03	0.22

Data is represented as mean ± S.D. (n=3).

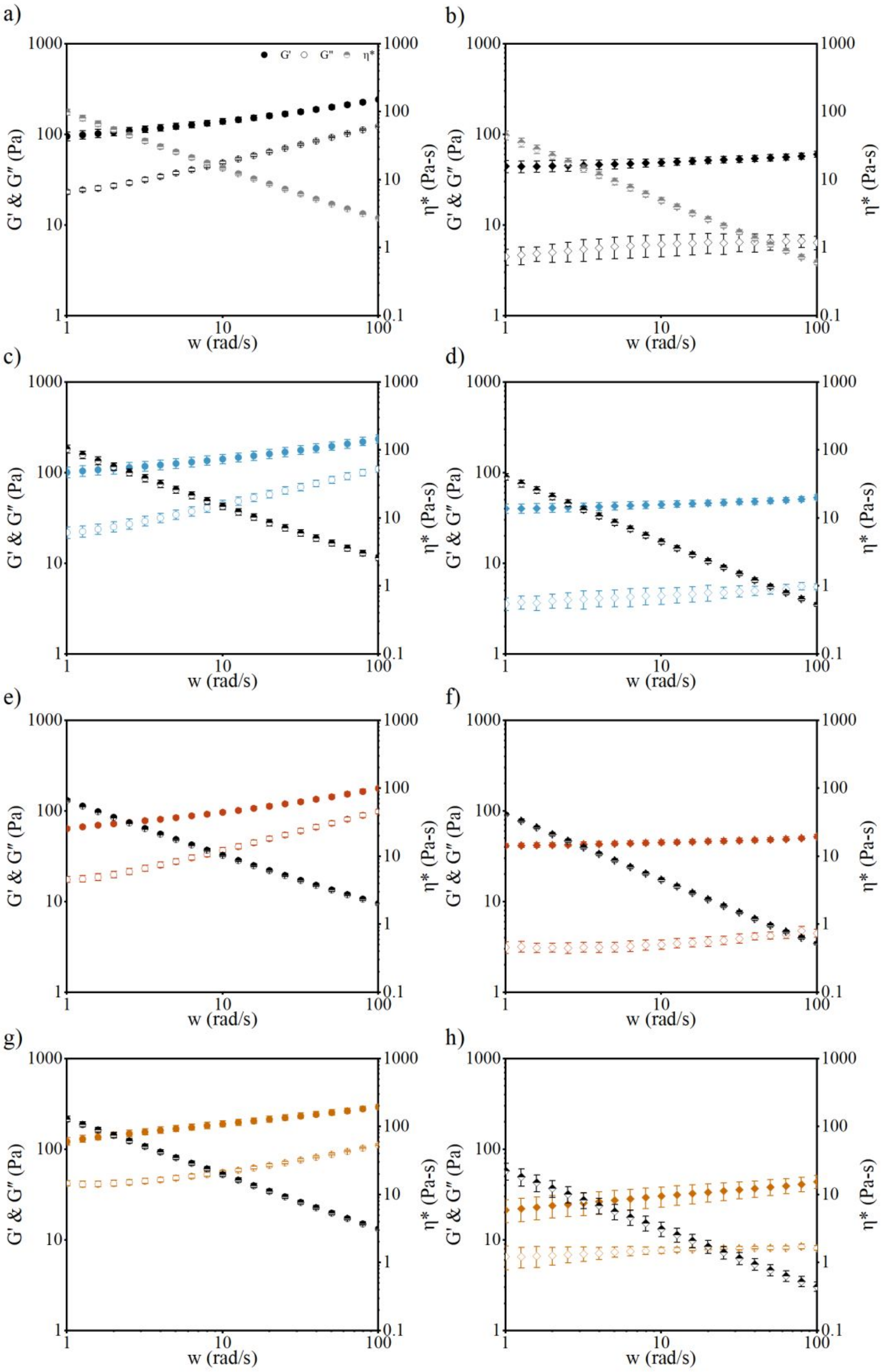

Figure S4. *Frequency sweep of hydrogels as polymerized (left column) and swollen purified (right column): $pN_{53}D_{47}$ a.) AP and b.) Spure; $pGal_{16}N_{45}D_{39}$ c.) AP and d.) Spure; $pGlc_{22}N_{41}D_{37}$ e.) AP and f.) Spure; $pGlc_{39}N_{39}D_{22}$ g) AP and h.) SPure. Rheological measurements were performed at 21 °C.*

Data is represented as mean ± S.D. (n=3)

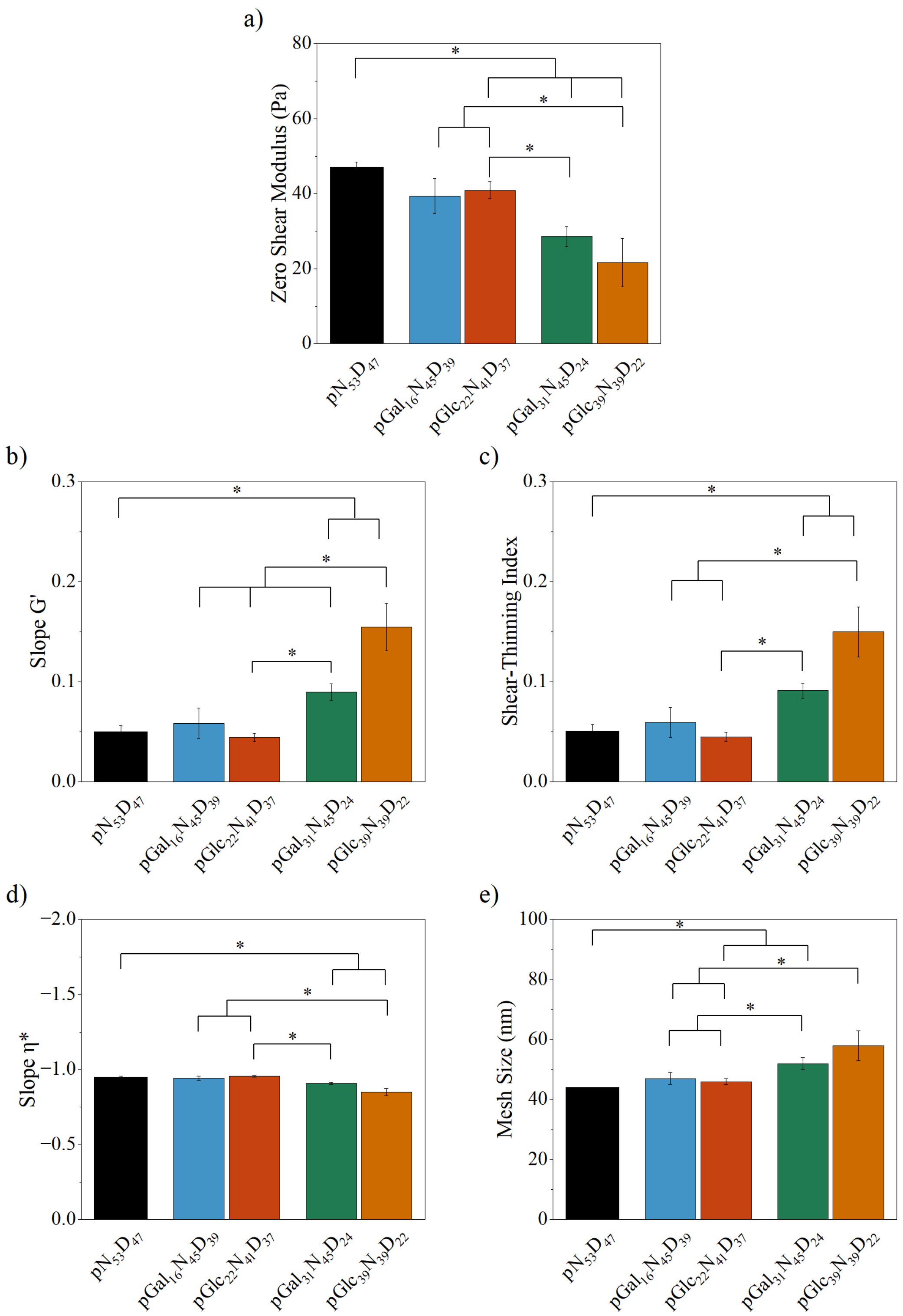

Figure S5. Statistical analysis of rheological data for Spure hydrogels a) G'_0, b) Slope of G' vs frequency curve, c) Shear-thinning index, d) Slope η*, e) Mesh size.
Asterisks above bars represent statistically significant differences in means (t-test, $p < 0.05$). Data is represented as mean ± S.D. (n=3).

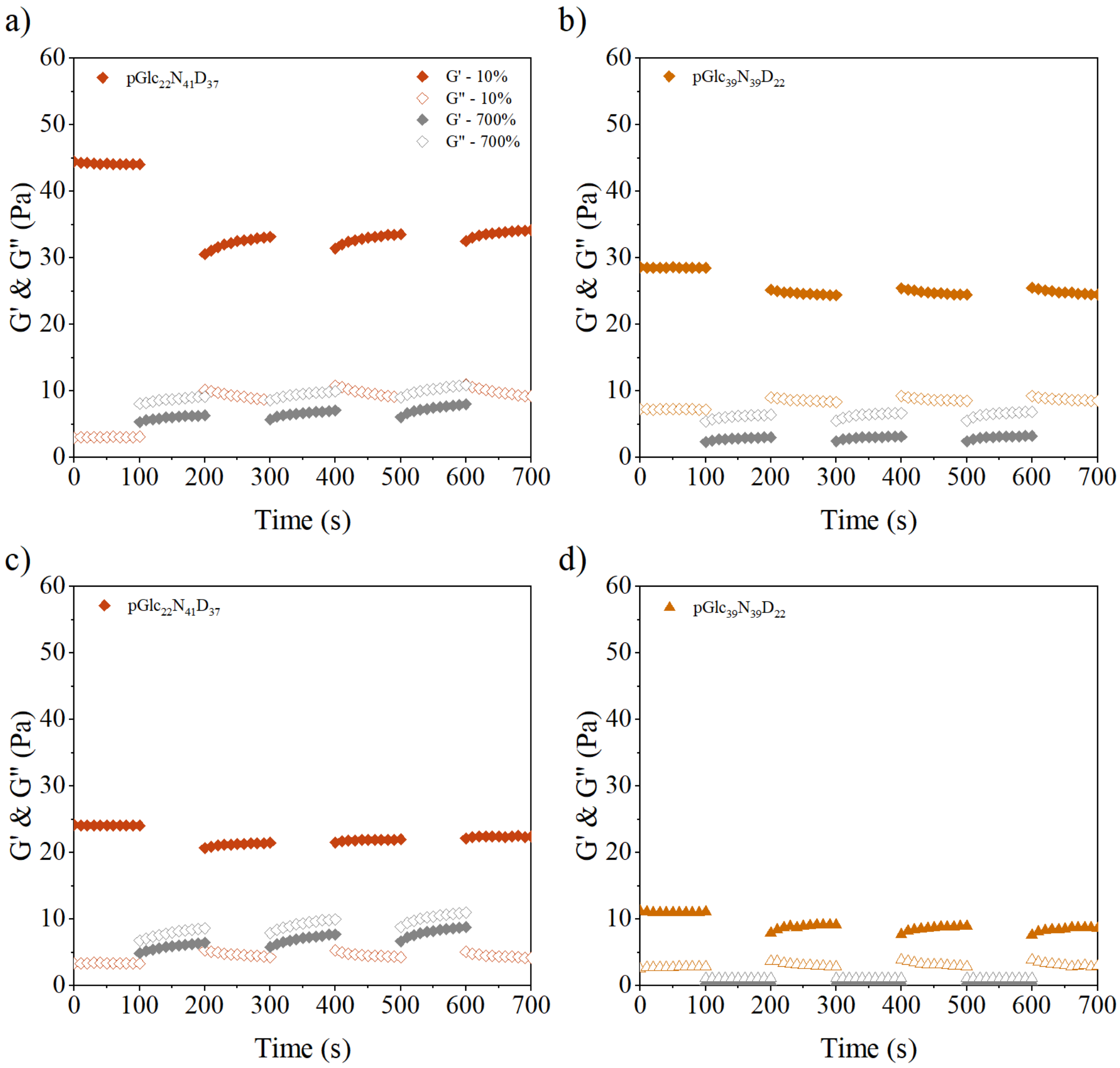

Figure S6. *Multicycle alternating step strain experiments of SPure hydrogels (top row) and pH adjusted hydrogels (bottom row): pGlc$_{22}$N$_{41}$D$_{37}$ a.) SPure and c.) Spure_pH3; pGlc$_{39}$N$_{39}$D$_{22}$ b.) SPure and d.) Spure_pH3. Rheological measurements were performed at 21 °C.*

Table S3. *Rheological properties of SPure_pH3 hydrogels. Rheological measurements were performed at 21 °C.*

Hydrogel	G_0' (Pa)	Mesh size (nm)	Slope of G'	Shear-thinning index	Slope of η*
$pN_{53}D_{47}$	23 ±3	57 ±2	0.13 ±0.02	0.12 ±0.01	-0.89 ±0.01
$pGal_{16}N_{45}D_{39}$	22 ±4	57 ±4	0.11 ±0.03	0.10 ±0.02	-0.90 ±0.02
$pGlc_{22}N_{41}D_{37}$	19 ±3	60 ±3	0.11 ±0.02	0.10 ±0.02	-0.90 ±0.02
$pGal_{31}N_{45}D_{24}$	15 ±1	65 ±11	0.14 ±0.01	0.14 ±0.01	-0.86 ±0.01
$pGlc_{39}N_{39}D_{22}$*	8	79	0.15	0.14	-0.86

Data is represented as mean ± S.D. (n=3).
*Data was not produced in triplicate (n=1).

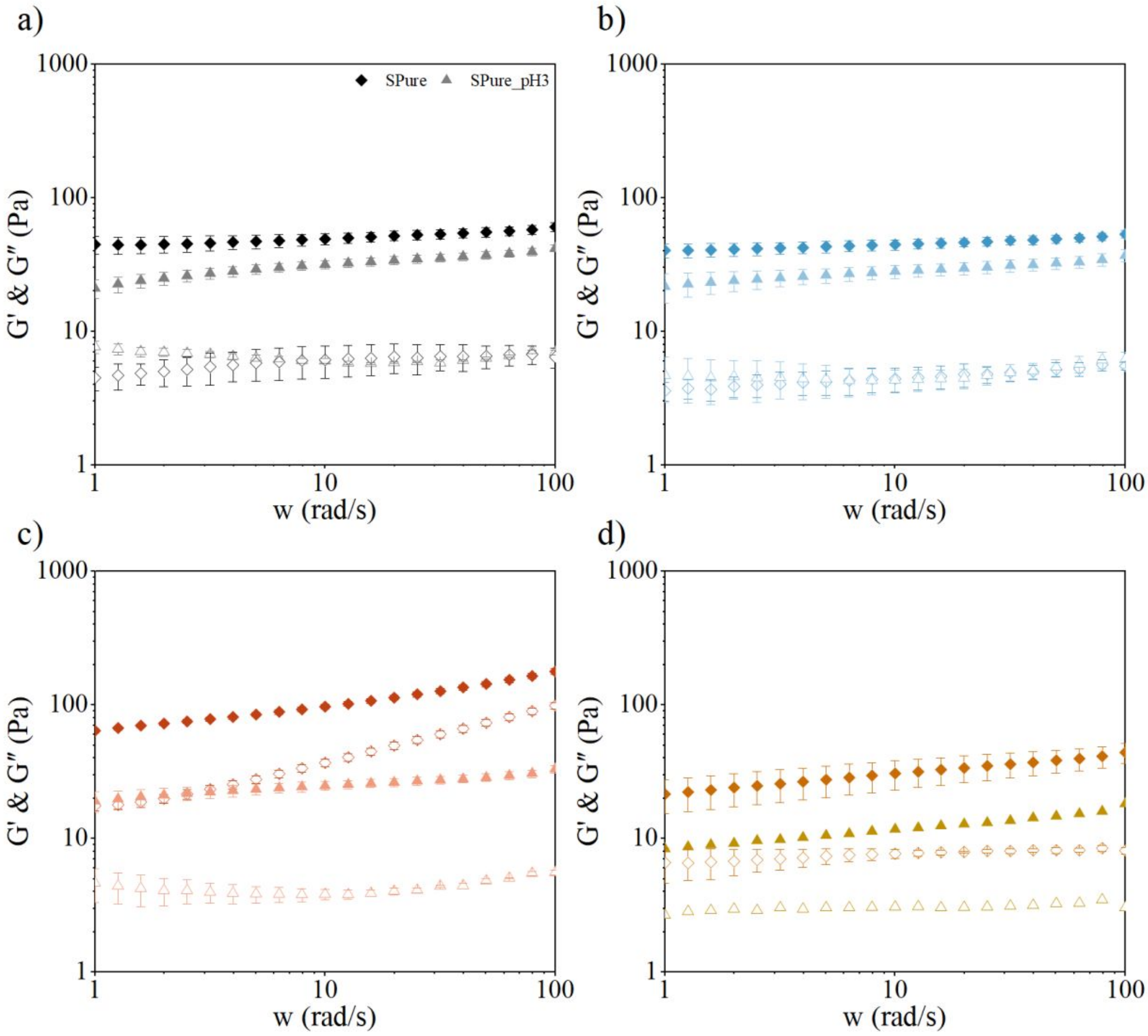

Figure S7. *Storage modulus comparison of SPure and SPure_pH3 hydrogels a.) $pN_{53}D_{47}$ b.) $pGal_{16}N_{45}D_{39}$ c.) $pGlc_{22}N_{41}D_{37}$ d.) $pGlc_{39}N_{39}D_{22}$. Rheological measurements were performed at 21 °C.*

Data is represented as mean ± S.D. (n=3).

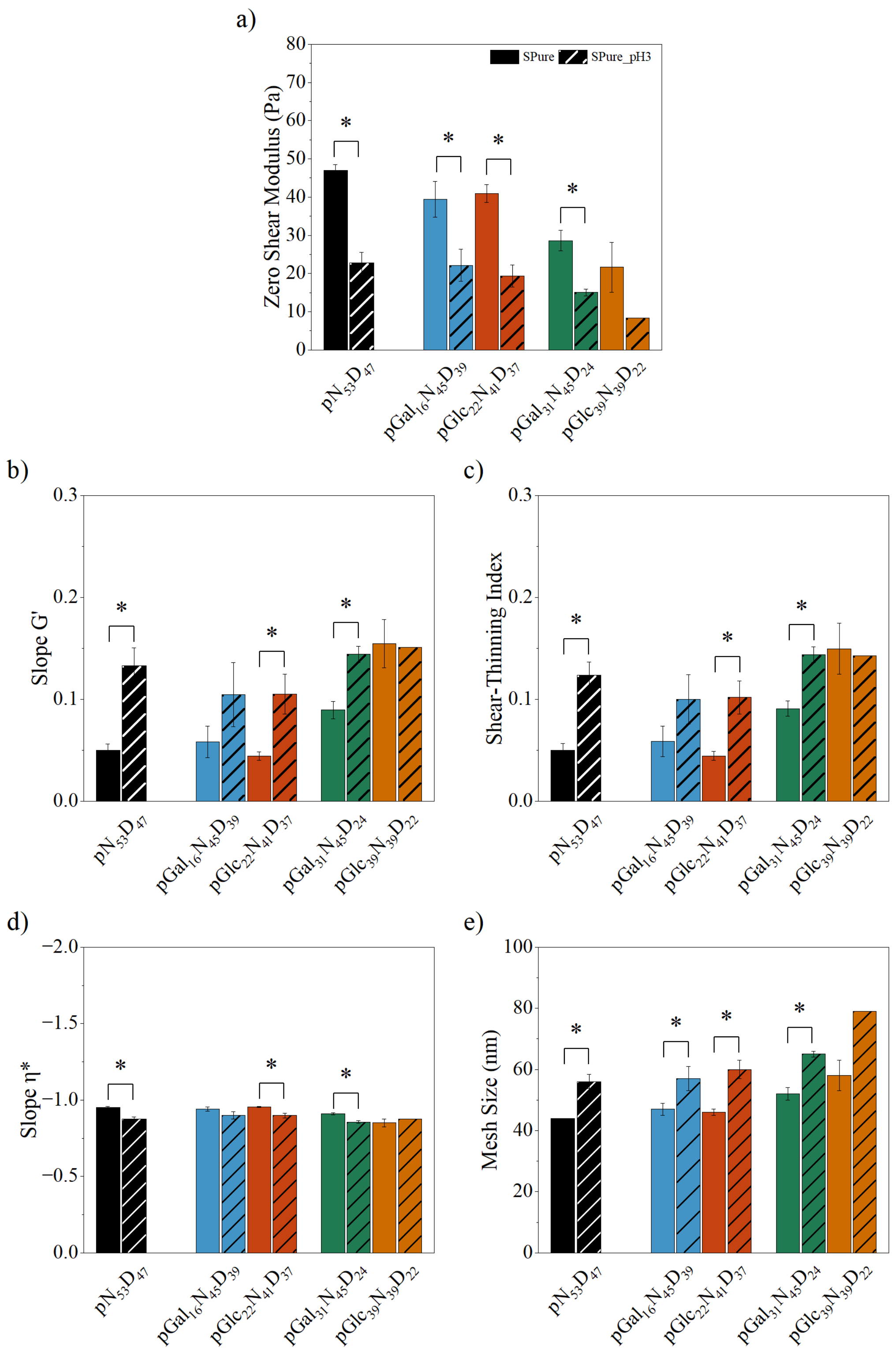

Figure S8. Statistical analysis of rheological data for Spure vs Spure_pH3 hydrogels a) G'_0, b) Slope of G' vs frequency curve, c) Shear-thinning index, d) Slope η*, e) Mesh size.
Asterisks above bars represent statistically significant differences in means (t-test, $p < 0.05$). Data is represented as mean ± S.D. (n=3).

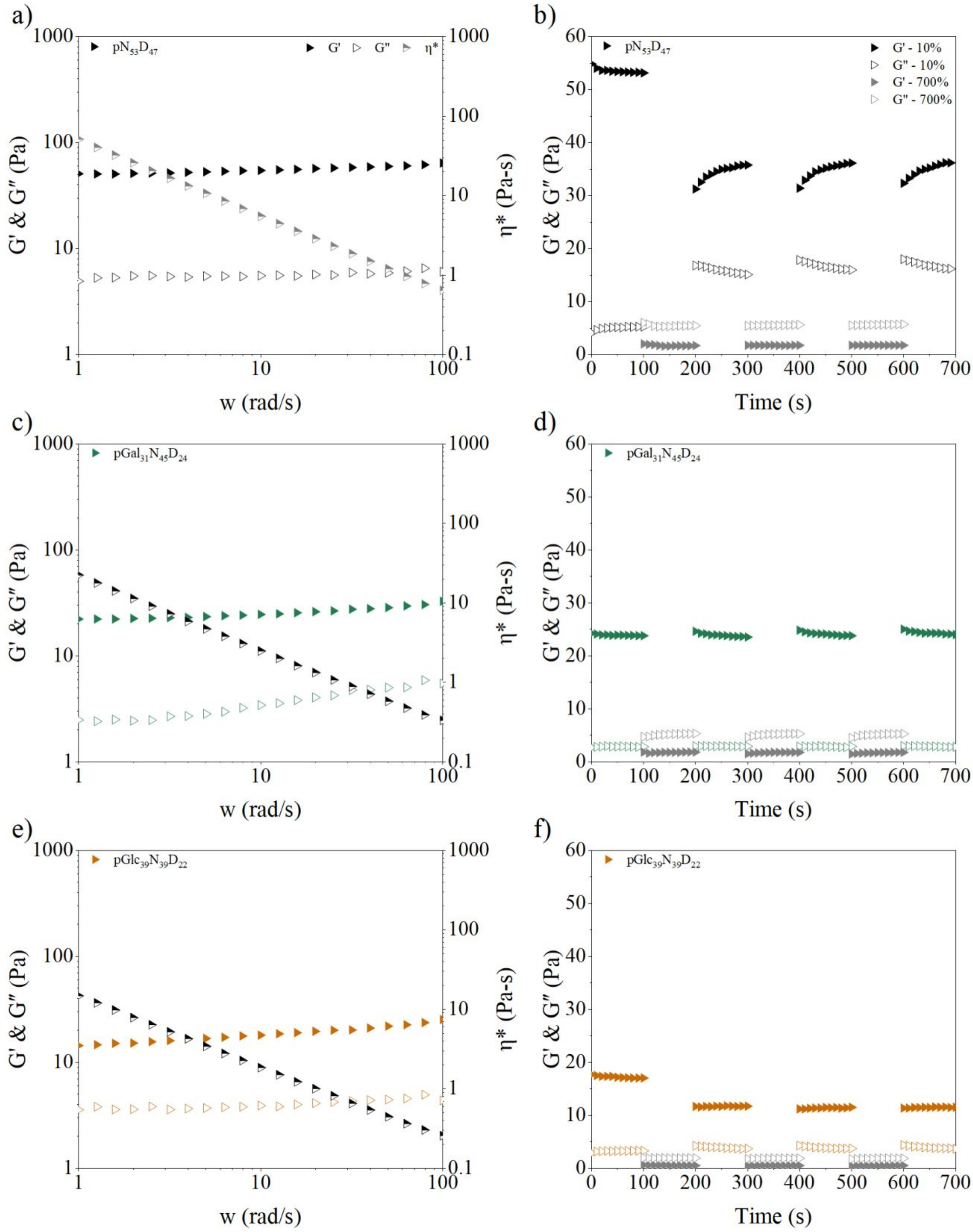

Figure S9. *Frequency sweep (left column) and multicycle alternating step strain experiments (right column) of SPure_RNA hydrogels: pN53D47 a.) and b.); pGal31N45D24 c.) and d.); pGlc39N39D22 e) and f). Rheological measurements were performed at 21 °C.*

Table S4. *Cumulative release of MO in SPure and Spure_pH3 hydrogels.*

Hydrogel	Cumulative Release 24 h (%)		Cumulative Release 120 h (%)	
	SPure	SPure_pH3	SPure	SPure_pH3
$pN_{53}D_{47}$	13 ±1	3 ±1	20 ±3	9 ±4
$pGal_{16}N_{45}D_{39}$	15 ±0	9 ±7	28 ±2	25 ±11
$pGlc_{22}N_{41}D_{37}$	15 ±1	6 ±2	31 ±2	12 ±3
$pGal_{31}N_{45}D_{24}$	27 ±1	12 ±0	34 ±2	39 ±2
$pGlc_{39}N_{39}D_{22}$	51 ±6	45*	58 ±6	71*

Data is represented as mean ± S.D. (n=3)
*Data is not produced in triplicate.

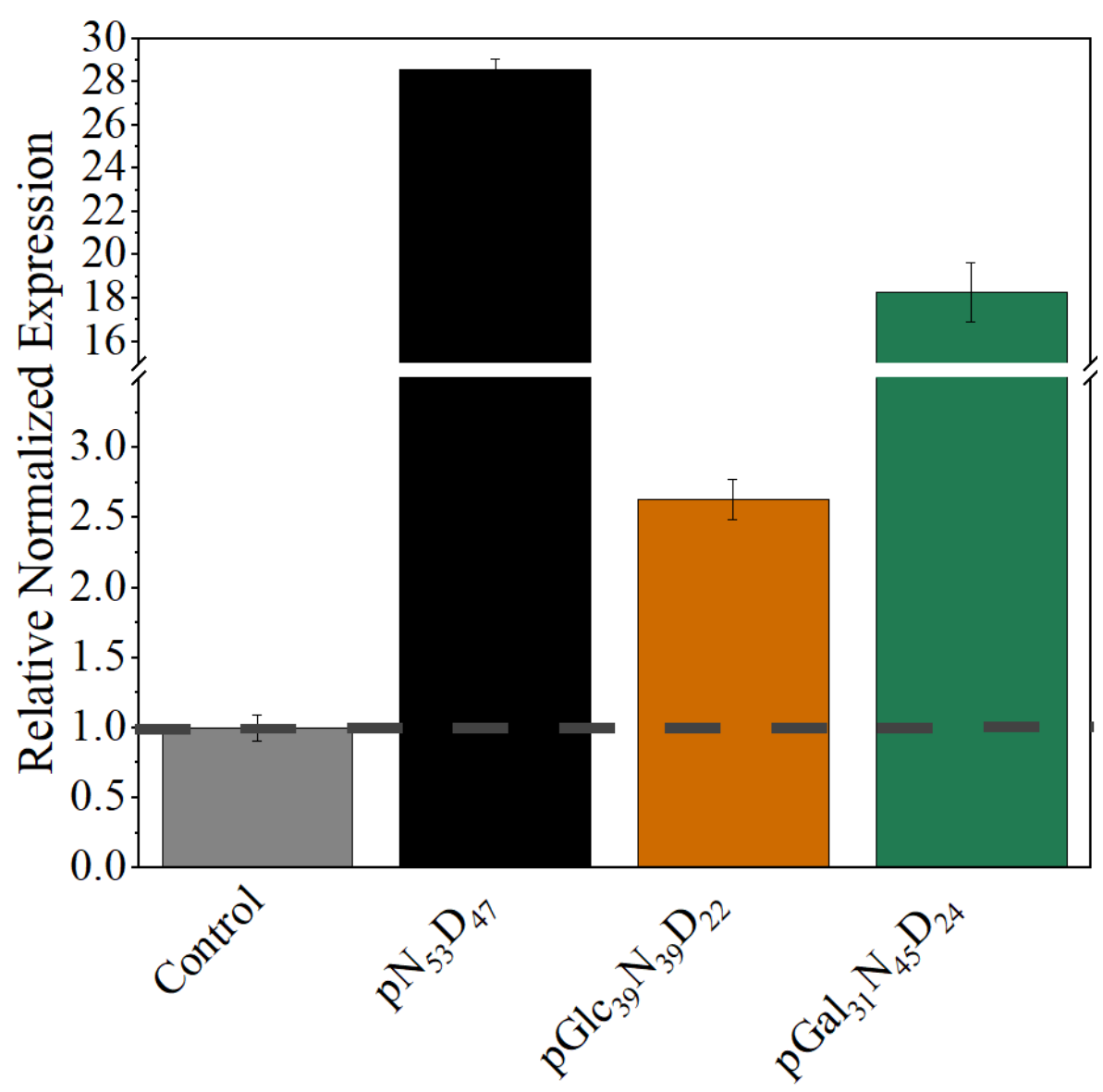

Figure S10. *Knockdown of Galectin alters gene expression in the salivary tissues of partially blood-fed ticks.*
Data represent mean ±SEM. Expression levels were normalized to the reference gene *Amblyomma americanum β-actin.*
